# A framework to score the effects of structural variants in health and disease

**DOI:** 10.1101/gr.275995.121

**Published:** 2022-04

**Authors:** Philip Kleinert, Martin Kircher

**Affiliations:** 1 Berlin Institute of Health (BIH) at Charité–Universitätsmedizin Berlin, 10117 Berlin, Germany;; 2 Institute of Human Genetics, University Medical Center Schleswig-Holstein, University of Lübeck, 23562 Lübeck, Germany

## Abstract

Although technological advances improved the identification of structural variants (SVs) in the human genome, their interpretation remains challenging. Several methods utilize individual mechanistic principles like the deletion of coding sequence or 3D genome architecture disruptions. However, a comprehensive tool using the broad spectrum of available annotations is missing. Here, we describe CADD-SV, a method to retrieve and integrate a wide set of annotations to predict the effects of SVs. Previously, supervised learning approaches were limited due to a small number and biased set of annotated pathogenic or benign SVs. We overcome this problem by using a surrogate training objective, the Combined Annotation Dependent Depletion (CADD) of functional variants. We use human- and chimpanzee-derived SVs as proxy-neutral and contrast them with matched simulated variants as proxy-deleterious, an approach that has proven powerful for short sequence variants. Our tool computes summary statistics over diverse variant annotations and uses random forest models to prioritize deleterious structural variants. The resulting CADD-SV scores correlate with known pathogenic and rare population variants. We further show that we can prioritize somatic cancer variants as well as noncoding variants known to affect gene expression. We provide a website and offline-scoring tool for easy application of CADD-SV.

In the light of recent advances in the field of structural variant (SV) detection and the study of regulatory domain architectures, phenotypic effects of SVs in humans moved into the focus of research (Lupiáñez et al. 2015; Sudmant et al. 2015; Chiang et al. 2017; Collins et al. 2020; Ebert et al. 2021). SVs can be deletions, duplications, insertions, translocations, or inversions and often span multiple kilobases of sequence in the genome. Due to their size, they have the potential to cause significant phenotypical effects and are therefore relevant for clinical genetics (Rodriguez-Revenga et al. 2007; Lupiáñez et al. 2015; Chiang et al. 2017; Spielmann et al. 2018). Although SVs affecting the expression of whole genes or exons are still the research focus, effects of noncoding DNA sequence alterations are of high interest. These variants are especially hard to predict, as our understanding of such regions lags behind coding annotations (Gloss and Dinger 2018). In comparison to pathogenic variants (e.g., frameshift mutations or disruption of transcription factor binding) caused by single nucleotide variants (SNVs), structural variants have a higher potential to affect the regulatory architecture of the genome. Thus, the functional characterization of SVs may help us to understand unexplained disease phenotypes and contribute to our understanding of regulatory mechanisms.

Recent advances in the study of regulatory genome architectures provided evidence along these lines and shed light on previously unexplained human disease conditions (Lupiáñez et al. 2015, 2016). The most relevant examples are improved Hi-C protocols to study genome architecture (Lieberman-Aiden et al. 2009), the experimental annotation of enhancers and enhancer-promoter links (Gasperini et al. 2020), mapping of multiple epigenetic features across many cell types (The ENCODE Project Consortium 2012) but also methods to test the regulatory potential of sequences in high-throughput (Inoue and Ahituv 2015; Nguyen et al. 2016; Santiago-Algarra et al. 2017; Kircher et al. 2019). All these advances provide a basic understanding of topological domain structures, regulatory elements, and other fundamental mechanistic insights like enhancer hijacking (Haller et al. 2019; Helmsauer et al. 2020). However, wider understanding of how SVs link to phenotypic alterations and therefore human diseases remains poor.

SV identification and annotation lags behind SNV and small insertion/deletion (indel) annotation, as SVs often exceed the size of common read-lengths, are difficult to align, fall within repetitive regions, or can be of complex structure (Cameron et al. 2019). In addition, various factors may contribute to pathogenicity or molecular effect in these regions as structural rearrangements can affect primary gene structure, chromatin architecture, DNA accessibility, and tissue-specificity of regulatory elements and genes. Further, the putatively different mechanisms of phenotypic effects of deletions compared to insertions or duplications complicates a generalized approach for variant effect prediction, as the effect can be mediated by copy number alterations of redundant or unique genomic sequence, positional effects, or rendering functional DNA dysfunctional. Capturing all possible disease-relevant mechanisms mediated by structural variants remains challenging.

Although various tools are available for ranking SNVs and indels, very few tools can score structural variants. Therefore, it remains very difficult to assess SV effects on phenotype and disease, with many different ad hoc approaches being applied. Existing tools like SVScore (Ganel et al. 2017) or TAD-fusion (Huynh and Hormozdiari 2019) focus on individual features such as the presence of deleterious SNVs (mostly in coding regions) that are overlapping the SV, or focus specifically on boundary element reshuffling by a novel SV, respectively. AnnotSV (Geoffroy et al. 2018) annotates the structural variant and categorizes pathogenicity depending on overlap with known pathogenic SVs. SVFX (Kumar et al. 2020) provides a framework for training specific models but does not allow the direct application to novel variants. At this stage, no tool combines ease of use with a comprehensive set of annotations, including the prioritization of disease effects from genome architecture alterations.

Further, SV data sets of sufficient size and curation that can be used to apply machine learning approaches for the identification of relevant annotations or for their integration are not easy to obtain. Clinically relevant SV sets (Landrum et al. 2018), that is, pathogenic and benign variants, are small in number, biased toward very large SVs, and tend to overlap well-studied disease genes. In this study, we aim to add a novel machine learning approach (CADD-SV) to score the effects of SVs by choosing an unbiased and sufficiently large training data set derived from species differences that is capable of differentiating between functional and nonfunctional SVs in the human genome. To validate this new approach, we apply CADD-SV to distinguish common SVs from annotated disease-causing variants and to identify functional variants on independent data sets of germline and somatic SVs. Our tool can be used to highlight disease-causing SVs in supposedly healthy individuals—for instance, recessive pathogenic variants in the gnomAD-SV cohort (Collins et al. 2020)—and allows prioritization of regulatory, noncoding variants like expression Quantitative Trait Loci (eQTLs) or variants under natural selection. We design CADD-SV as a web service as well as a standalone tool for easy application and interpretation of novel SVs.

## Results

### A large and unbiased training data set

Machine learning methods strongly rely on the quality of training data sets to yield meaningful predictions. Using clinical databases such as ClinVar or the Human Gene Mutation Database (HGMD) to curate an annotated training data set is challenging for SNVs or small indels, where a careful matching of pathogenic and benign variants in genomic regions and effect classes is required (Huang et al. 2017; Rentzsch et al. 2019). This seems currently impossible for SVs. The ClinVar data set (Landrum et al. 2018) is very sparse for SVs; that is, only a few (3262 deletions, 82 duplications, and 78 insertions) and mostly very large SVs (mean size of 106 kb for deletions) are being annotated. This is insufficient for an insightful training data set, especially as population-derived SVs are much smaller in genomic size (mean of 7.4 kb). Further, when compared to large population SV sets (Collins et al. 2020), strong biases toward high effect variants and clustering around well-studied genes are apparent (Supplemental Fig. 1). Therefore, we opt for an unbiased evolutionary set of SVs obtained from comparisons in the great ape lineage (Kronenberg et al. 2018). A key strength of this approach is that the model is trained on a larger training set of 19,113 deletions and 26,823 insertions and duplications that does not suffer from the ascertainment bias inherent in curated sets (Supplemental Fig. 1).

**Figure 1.**
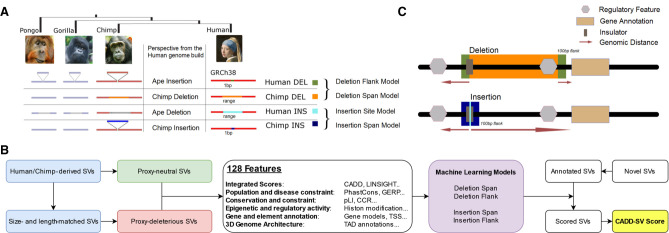
Workflow and training data sets of the CADD-SV framework. (*A*) Proxy-neutral training data set of CADD-SV. Human- and chimpanzee-derived structural variants (SVs) are considered to be neutral or beneficial if they reached fixation. Therefore, previously identified human- and chimpanzee-derived SVs (Kronenberg et al. 2018) are used as a proxy-neutral training data set. (*B*) CADD-SV workflow. Size- and length-matched simulated variants are used as a proxy-deleterious training data set. Next, various informative features are annotated and transformed (see Methods; Supplemental Table 1) across span or flank of the variants to train multiple random forest classifiers. Models are used to score user-provided (novel) SVs. For this purpose, variants are annotated, features transformed, and models applied. The maximum value of the flank and span model scores is used as the raw model score. Further, a Phred transformation of the relative rank of the score among gnomAD-SVs provides an easy interpretation of the CADD-SV score. (*C*) Depiction of implementation of the four models generated from the proxy-neutral and proxy-deleterious variant sets. Whereas deletion of a novel sequence provides information about the deleted sequence in the human genome build, the insertion model relies on the site of integration. Therefore, flanking regions to the SVs are taken into account.

This is motivated by the Combined Annotation Dependent Depletion (CADD) framework, an approach that has proven powerful in the interpretation of SNVs and short indels (Kircher et al. 2014). In CADD-SV, we assume that millions of years of purifying selection removed SVs that are deleterious, that is, have a negative impact on human or chimpanzee reproductive success. Thus, fixed SVs in humans or chimpanzees can be classified as proxy-neutral. In contrast, variants of the same size randomly drawn from the human genome are likely to contain a significant number of deleterious variants by chance. Although many of the random variants will be neutral, an unknown but considerable fraction would likely be deleterious. For simplicity, we refer to these variants as proxy-deleterious. The contrast between the proxy-neutral and proxy-deleterious variant sets, that is, the relative paucity of deleterious, phenotypically influential genome alterations in the proxy-neutral set and the resulting differences in their annotation features, is the core characteristic of what we then model as SV deleteriousness (Fig. 1A).

### Annotating structural variants

We wanted to integrate diverse annotations into predictive, genome-wide models for identifying structural variants of phenotypic effect. Although many annotations are readily available for SNVs, informative and computationally efficient statistics need to be created to summarize annotations over the span of SVs. Further, distance measures can retain information about the vicinity of the impacted DNA sequence. For this purpose, we developed an automated SV annotation pipeline (Fig. 1B) using the workflow management system Snakemake (Köster and Rahmann 2012) that combines BEDTools (Quinlan and Hall 2010) and tabix (Li 2011) with customized bash and R scripts. We integrated not only coding information such as gene models but also a wide variety of regulatory annotation retrieved from ENCODE (The ENCODE Project Consortium 2012), such as histone modifications or DNA accessibility. In addition, we made use of functional and evolutionary scores (Siepel et al. 2005; Davydov et al. 2010; Huang et al. 2017; Rentzsch et al. 2019) as well as information about the 3D architecture of the genomic region derived from Hi-C experiments (Schmitt et al. 2016; Calandrelli et al. 2018; Schwessinger et al. 2020).

All SVs are annotated over the full span of the event as well as 100 bp up- and downstream (Fig. 1C). For insertions, the span of novel SVs only contains the site of integration, and CADD-SV does not derive features from the inserted sequence. Whereas deletions directly remove putatively functional sequence, insertions and duplications interfere with molecular function by integration of additional sequence, for example, disrupting regulatory interactions by increasing distance or introducing frameshifts into coding sequence. We incorporate this in the CADD-SV modeling by deriving features from the deleted sequence (span), annotating the context of the SV (flank), and including distance features in the model (Fig. 1B,C). Across SV ranges, we mostly annotate max values, mean values, and the amount of high-impact values above the top 90th percentile of an annotation. Additionally, span and flank models use genomic distances to certain feature coordinates (e.g., genes, exons, and enhancers). All features and their transformation are described in Supplemental Table 1. To ease later interpretation of feature impact, all features are *Z-*score-transformed (mean 0, standard deviation of 1) using the annotation value distributions of the same type of SV from healthy individuals reported in gnomAD (Collins et al. 2020). This transformation serves primarily the interpretability of the model and does not negatively affect model training, as the same transformation is applied for both training class labels.

### Modeling and holdout set performance

SV-mediated pathogenicity depends on the type of SV. We implement separate models for deleted (DEL), inserted (INS), or duplicated (DUP) sequence. Currently, due to the lack of training data for inversions and translocations, we cannot train models for these variant types. Using the described training data sets, we train four types of models (Fig. 1A,B). We train models of human-derived deletion (human DEL) and insertion events (human INS) against respective sets of equally sized events drawn across the genome. Further, models based on chimp insertion (chimp INS) and deletions events (chimp DEL) are trained. Here, we project the events onto the human reference sequence and use the human annotations. Whereas the human events are also manifested in the human reference, the chimp events allow us to use human annotation unimpaired by an actual SV event. Hence, chimp DEL models are similar to how we would score new events observed in an individual's genome aligned to the human reference sequence. In contrast, no annotation for human-derived deletions can be obtained over the span of the deletion, as experimental readouts and conservation score are not available for the missing sequence. Similarly, chimp INS provides an insertion model based on events that did not impair human annotations or biochemical readouts.

To score novel SVs in the human genome, we exploit this relationship by training the span of novel deletions with the chimp DEL set and train the sequence 100 bp up- and downstream of the breakpoints using the human DEL set. As the inverse applies for insertions and duplications, that is, chimpanzee insertions do not span sequence in the human genome build whereas human-derived insertions do, we use the chimp INS set for the insertion site and the human INS set for the up- and downstream sequence. Duplications are scored using the full sequence span of the duplicated locus, hence using the chimp DEL model for the span and human INS model for the up- and downstream sequence. The final score is calculated from the maximum (more deleterious) value of both models.

**Figure 2.**
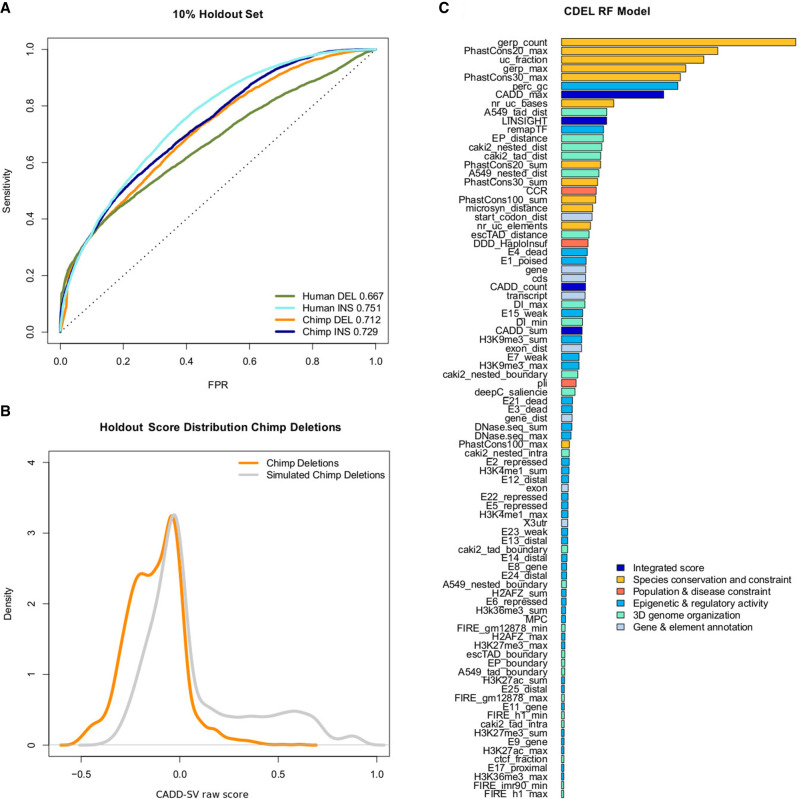
Performance of random forest models trained on proxy-deleterious and proxy-benign SVs. (*A*) All models show a nonrandom separation of the two classes in a random 10% holdout. Performance is measured as sensitivity over false positive rate (FPR). Note that all training data sets contain a high amount of mislabeled SVs, as a majority of proxy-deleterious SVs is likely to be neutral. (*B*) Model predictions of the chimpanzee deletion model are shifted toward high-impact SVs in the simulated set of chimpanzee deletions. (*C*) Representation of feature importance in the chimpanzee deletion random forest model. Note that proxy-pathogenic and proxy-benign sets are length-matched and that length is not used as an explicit feature. Most important contributions come from species conservation (e.g., GERP, phastCons) but also from integrated scores (i.e., CADD or LINSIGHT). Epigenetic features as well as 3D genome architecture features, such as the Directionality Index derived from Hi-C data, also contribute to the most informative features of the models. For a full list of features and explanation of their naming, see Supplemental Table 1.

We trained both logistic regression models as well as random forest models. We note that the latter show increased holdout performance as well as validation set performance (Supplemental Fig. 2), and we only describe the random forest models here. We opted for measuring validation performance on a holdout rather than cross validation, as the choice of training data allows for a sufficiently large training set. The holdout shows that all four model types differentiate between the proxy-benign and proxy-deleterious sets (Fig. 2A). Considering the anticipated mislabeling in our training data, specifically in the randomly drawn SVs as described above, the holdout performance will, however, not be representative for our models’ performance in scoring actual pathogenic versus benign variants. Here, we only look for a nonrandom model performance and the relative ranking of the INS, DEL, and DUP models. The model score distribution for the holdout data is available in Figure 2B for the proxy-deleterious and proxy-benign SV sets. We see a significant shift with a bimodal distribution in the proxy-deleterious variants, with the smaller mode corresponding to the potentially pathogenic variants in the randomly drawn set.

For better interpretation, we also provide a Phred-scaled transformation of the model score relative to a healthy population cohort, that is, a log_10_ score derived from the proportion of variants with a greater or equal score in the genomAD-SV set. The CADD-SV scores on the Phred scale range from 0 (potentially benign) to 48 (potentially pathogenic), indicating the position of the novel variant within the gnomAD-SV score distribution. For example, a score above three corresponds to the top 50%, 10 corresponds to the top 10%, 20 to the top 1%, and 30 to the top 0.1% of scores observed from gnomAD-SV.

### Feature contributions

We analyzed feature contributions in our random forest models using the R package randomForest (Liaw and Wiener 2002). To ease interpretation, we categorized model features into six groups (“Integrated scores”, “Species conservation and constraint”, “Population and disease constraint”, “Epigenetic and regulatory activity”, “3D genome organization”, “Gene and element enrichment”) (Supplemental Table 1). Models benefit highly from features in the groups of “Species conservation and constraint” (including GERP, phastCons, phyloP scores) and “Integrated scores” (i.e., summaries of CADD SNV and LINSIGHT scores) in differentiating between the contrasted SV sets. Regulatory annotations as well as 3D genome architecture features contribute to a smaller extent but are present within the top 20 most important features of all models (e.g., ReMap transcription factor occupancy, TAD annotations, enhancer-promotor links, and ChromHMM states). Distance features (such as distance to coding sequence) are particularly prevalent in the human DEL flank model where, for a reference altered by the deletion event, these features become informative. Major feature contributions of the chimp DEL model are presented in Figure 2C; for all models, feature importance is available in Supplemental Figures 3–6.

### Independent validation data sets

**Figure 3.**
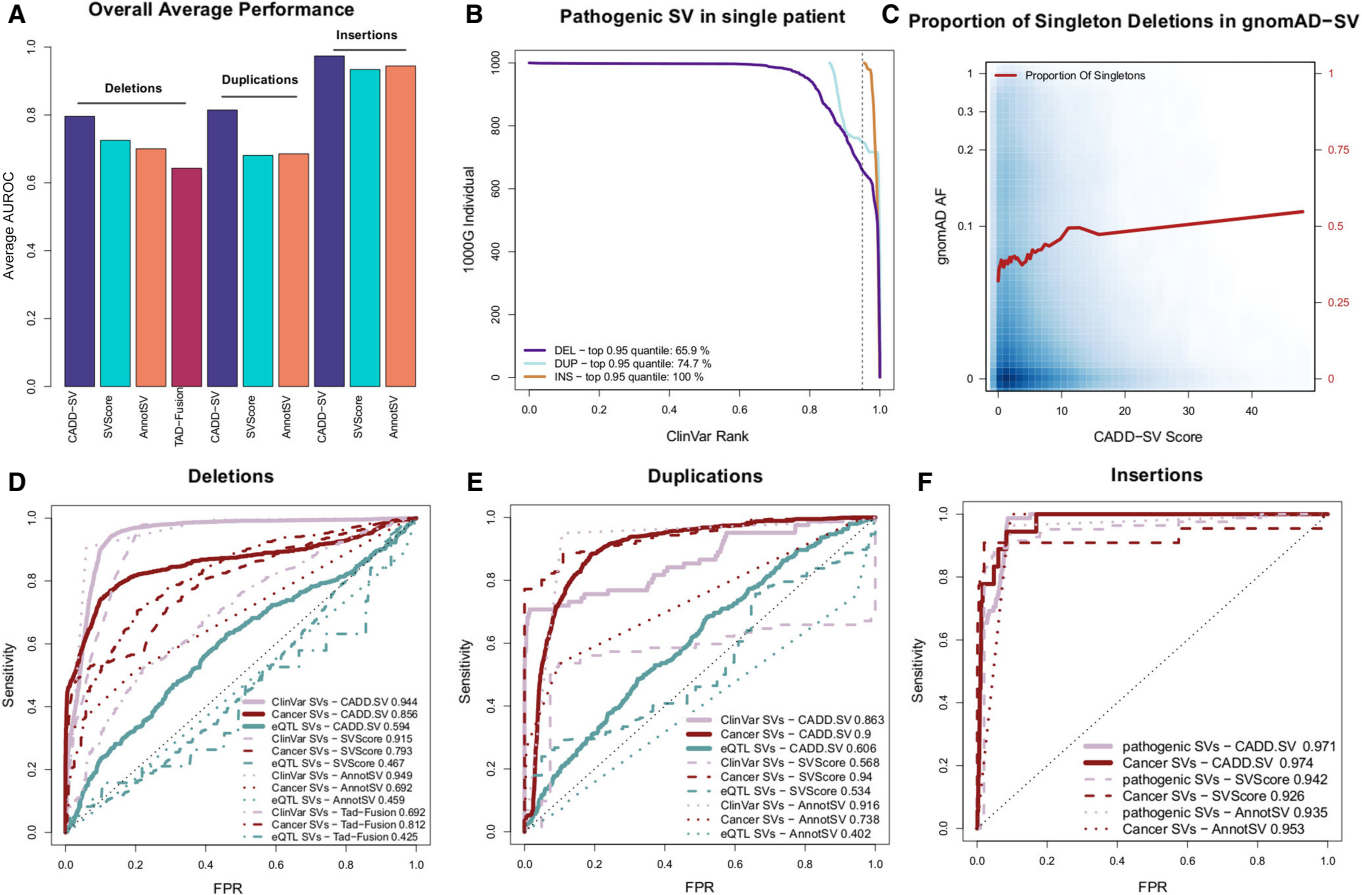
Validation set performance of the random forest models. (*A*) Summary of the performance of CADD-SV scores compared to SVScore, AnnotSV, and TAD-fusion scores across three validation sets (pathogenic variants, cancer variants, and putative eQTL SVs) for deletions, duplications, and insertions. (*B*) Rank of ClinVar pathogenic SVs added to SVs of healthy individuals from the 1000 Genomes Project. CADD-SV prioritizes the pathogenic SVs over the other SVs in a single simulated patient, scoring pathogenic variants in the top fifth percentile of deletions, duplications, and insertions for 65.9%, 74.7%, and 100% of simulated variant sets, respectively. (*C*) CADD-SV score distribution as a function of gnomAD allele frequency. Higher CADD-SV values represent an increased likelihood to be deleterious. In the deleterious tail of the score distribution, there is an excess of singletons (shown in red; bin size 0.025), which hints at negative selection against deleterious deletions. (*D*–*F*) CADD-SV performance of various validation sets compared to common gnomAD SVs (AF ≥ 0.05). Performance is measured as sensitivity over false positive rate. CADD-SV is able to identify ClinVar pathogenic SVs (n = 3262 deletions, 82 duplications, and 78 insertions, pale red) as well as SVs reported in the ICGC cancer cohort (n = 52,677 deletions, 42,972 duplications, and 18 insertions, dark red) from common SVs in gnomAD. Further, CADD-SV can identify noncoding SVs that are associated with differences in gene expression (turquoise). CADD-SV scores (solid lines) are compared to SVScore (dashed lines), AnnotSV (dotted lines), and TAD-fusion (dashed and dotted lines) for deletions (*D*), duplications (*E*), and insertions (*F*).

To validate the general applicability of the framework, we use multiple lines of evidence (Fig. 3A) to substantiate the results of the holdout performance. We look at known pathogenic variants from ClinVar (Fig. 3B,D–F); we show that SVs occurring in healthy populations are under negative selection and therefore have high CADD-SV scores enriched for singleton events (Fig. 3C); and we analyze variants from the International Cancer Genome Consortium (Fig. 3D–F) and SVs affecting gene expression (Fig. 3D–F). Thereby, we show that CADD-SV can be used to prioritize both pathogenic germline and somatic structural variants.

#### Pathogenic germline variants

We collected pathogenic SVs from ClinVar (n = 3262 deletions, 82 duplications, and 78 insertions). To look at how CADD-SV prioritizes pathogenic variants among all SVs identified in single individuals (including rare and singleton events), we added each clinically characterized SV from ClinVar into sets of structural variants found in presumed healthy individuals from the 1000 Genomes Project (The 1000 Genomes Project Consortium 2015). We assessed the performance of CADD-SV by looking at the pathogenic variants’ rank among all observed SVs. We found that, in 65% of cases, the ClinVar deletion is within the top fifth percentile of all ranks (Fig. 3B). Clinically labeled insertions and duplications were also enriched among the top candidates. In 100% of individuals for insertions and 75% of individuals for duplications, these events fall within the top fifth percentiles.

Further, we contrasted the complete sets of pathogenic SVs from ClinVar with a matched number of common SVs from gnomAD (AF ≥ 0.05) (Fig. 3D–F). CADD-SV correctly identifies a vast majority of the known pathogenic SVs with an Area Under the ROC Curve (AUROC) of 0.944 for deletions (Fig. 3D). CADD-SV performs comparably to the existing tools SVScore (Ganel et al. 2017) with an AUROC of 0.915 and AnnotSV (Geoffroy et al. 2018) with an AUROC of 0.949. It outperforms TAD-fusion score (Huynh and Hormozdiari 2019), which has an AUROC of 0.692, but was primarily designed to detect 3D architecture alterations. Finally, we compared to StrVCTVRE (Sharo et al. 2020), which was designed to score exonic variants specifically and cannot score all of these variants. However, CADD-SV outperforms StrVCTVRE on prioritizing exonic ClinVar deletions from a background of exonic gnomAD-SV deletions (Supplemental Fig. 7).

#### Depletion of deleterious SV*s* in healthy populations

We assessed the distribution of CADD-SV scores in SVs from the gnomAD SV call-set. Allele frequency (AF) values are significantly decreased in the pathogenic tail of the CADD-SV score distribution compared to the benign tail (top/bottom fifth percentile CADD-SV scores, two-sided Wilcoxon rank-sum test, *P*-value < 10^−16^). We reason that CADD-SV is able to prioritize deleterious variants in healthy individuals, as these variants would be under negative selection and removed from the gene pool. Accordingly, the proportion of singleton deletions among the top fifth percentile CADD-SV scores (pathogenic tail) is 1.3 times higher than the average of the full SV set (Fig. 3C). This observation is striking for deletions but less pronounced in the insertion and duplication SV sets (Supplemental Fig. 8). We note that, in the top fifth percentile, 35% of deletions are coding variants classified as “Loss of Function” by gnomAD compared to 0.3% of variants scored in the remainder of the CADD-SV score distribution.

Further, the average deletion length is six times longer for the top fifth percentile compared to the rest of the distribution, suggesting that longer deletions are more likely to be functional, as they affect more sequence. However, short (<100 bp) and high scoring (top fifth percentile) deletions are 1.1 times more likely to be singletons compared to short deletions, suggesting that CADD-SV prioritizes SVs beyond length. In addition, we detect high-frequency deleterious variants in the pathogenic tail, speculating that these variants could be phenotypically functional variants and potentially beneficial for carriers.

#### Identifying somatic cancer variants

We assessed the performance of CADD-SV on somatic variants and the power to identify deleterious cancerogenous variants (n = 52,677 deletions, 42,972 duplications, and 18 insertions) using SV variants from cancer patients in the International Cancer Genome Consortium (Campbell et al. 2020) as well as insertions reported in Qian et al. (2017). We find an enrichment of SVs detected in cancer patients in the pathogenic tail of the distribution compared to SVs from a healthy cohort (two-sided Wilcoxon rank-sum test, *P*-value < 10^−16^). CADD-SV enriches the cancer-derived SVs from common gnomAD-SVs in an ROC curve analysis (AUROC values of 0.848, 0.933, and 0.975 for deletions, duplications, and insertions, respectively) (Fig. 3D–F), outperforming available tools on this task and supporting the claim that CADD-SV prioritizes functional somatic SVs.

#### Identifying expression-altering noncoding variants

To test the ability to prioritize functional variants beyond coding regions, we use a set of noncoding SVs known to alter the expression of genes. Here, we look at 387 deletions and 300 duplications that were shown to affect expression levels of nearby genes and are therefore considered eQTLs by the GTEx Consortium (Chiang et al. 2017). We compare them against common variants (AF ≥ 0.05) from gnomAD in an ROC curve analysis (Fig. 3D–F). Even though less pronounced compared to ClinVar or the cancer-derived SVs, CADD-SV is able to differentiate the two classes of SVs (AUROC 0.598 for deletions and 0.635 for duplications, respectively), outperforming existing methods SVScore (AUROC 0.467 for deletions and 0.534 for duplications), AnnotSV (AUROC 0.459 for deletions and 0.402 for duplications), and TAD-fusion score (AUROC 0.425 for deletions).

### Functional SVs in a healthy population cohort

**Figure 4.**
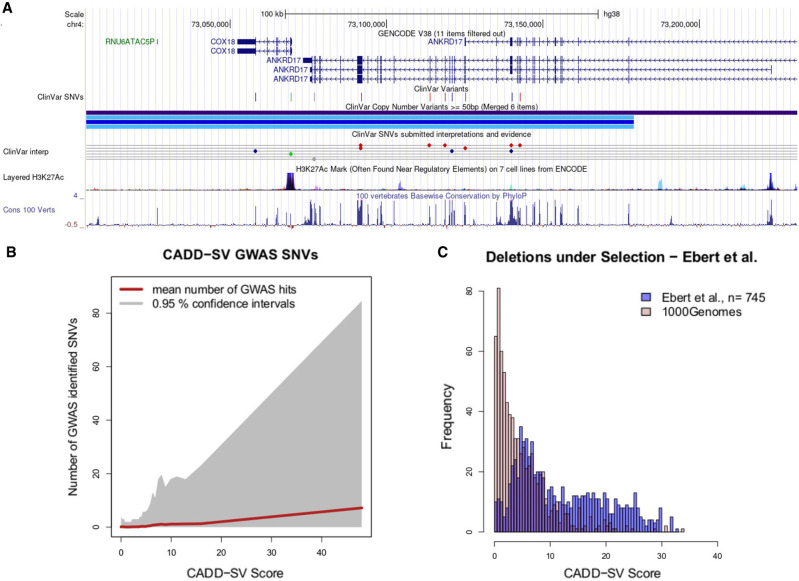
Prioritizing functional variants with CADD-SV. (*A*) Screenshot of UCSC Genome Browser tracks of a region (Chr 4: 73,004,055–73,231,324) deleted in one individual present in the gnomAD-SV cohort. Two genes are affected, with *ANKRD17* variants being reported as causal for the autosomal dominant Chopra-Amiel-Gordon syndrome (CAGS). Various pathogenic SNVs were identified within the gene body of *ANKRD17* and are marked in red in the UCSC ClinVar track. CAGS patients are characterized by developmental delay and moderate to severe intellectual disability. Further, various positions of this SV are highly conserved among 100 vertebrate genomes, contributing to CADD-SV's power of ranking it as a putatively deleterious variant. (*B*) Phred-scaled CADD-SV score distribution as a function of number of genome-wide association study-identified SNVs per deletion from gnomAD-SV. Especially among high scoring SVs, the average number of GWAS-associated SNVs increases drastically, suggesting functional variants in the pathogenic tail of the CADD-SV score distribution. (*C*) Scoring deletions under natural selection from Ebert et al. (2021). Shown are score distributions for the functional set (blue) against the same number of randomly drawn SVs from the 1000 Genomes Project. Note that we report Phred-scaled CADD-SV scores (log_10_ scale) with high values corresponding to high deleteriousness.

Variants reported in the gnomAD-SV database are considered largely benign, as this cohort consists of only healthy individuals, not excluding potential complex or late-onset diseases (Collins et al. 2020). Although being devoid of embryonal lethal variants, healthy data sets can contain pathogenic or haploinsufficiency variants that are expected to be under purifying selection and therefore rare in allele frequency. We showed that rare variants are strongly enriched in the most pathogenic tail of the CADD-SV distribution (Fig. 3C). We investigated the shortest (mean length of 225,336 bp) five top scoring variants (CADD-SV Phred score ≥ 35) and found all of them to be ultrarare (AF ≤ 0.0009), with three out of five being singletons (Supplemental Table 2). Further, three out of five variants overlap multiple ClinVar curated pathogenic variants, belonging to two autosomal recessive disease genes and one autosomal dominant disease gene. The two recessive diseases are Batten disease mediated by mutations in *CLN3* (see Supplemental Fig. 9; Munroe et al. 1997), and hearing loss mediated by mutations in *OTOA* (see Supplemental Fig. 10; Kim et al. 2019). The one autosomal dominant neurodevelopmental disorder is Chopra-Amiel-Gordon syndrome, mediated by mutations in *ANKRD17* (see Fig. 4A and Supplemental Fig. 11; Chopra et al. 2021).

Further, the tail of the CADD-SV pathogenic score distribution is strongly enriched in SVs containing genome-wide association study (GWAS)-identified SNVs, suggesting the presence of functional genomic regions (Fig. 4B). Containing a GWAS hit is not equal to being a potentially pathogenic SV, as many recorded associations are toward nondisease traits such as body height or longevity. However, it provides evidence that CADD-SV is able to prioritize functional stretches of sequence in the genome without using the GWAS catalog as an input itself. The top 10 gnomAD-SV variants contain an average of 265 GWAS-associated SNVs (Supplemental Table 3). Further, CADD-SV is able to prioritize an additional set of SVs (Ebert et al. 2021) under natural selection (Fig. 4C; Supplemental Fig. 12A,C), as well as SVs associated with expression changes (Supplemental Fig. 12B,D), with most Phred scores exceeding a value of 10 (top 10%) and many above 20 (top 1%) or even 30 (top 0.1%). This supports that CADD-SV is able to prioritize functional stretches of DNA genome-wide and beyond exonic regions.

### Interpreting structural variants

**Figure 5.**
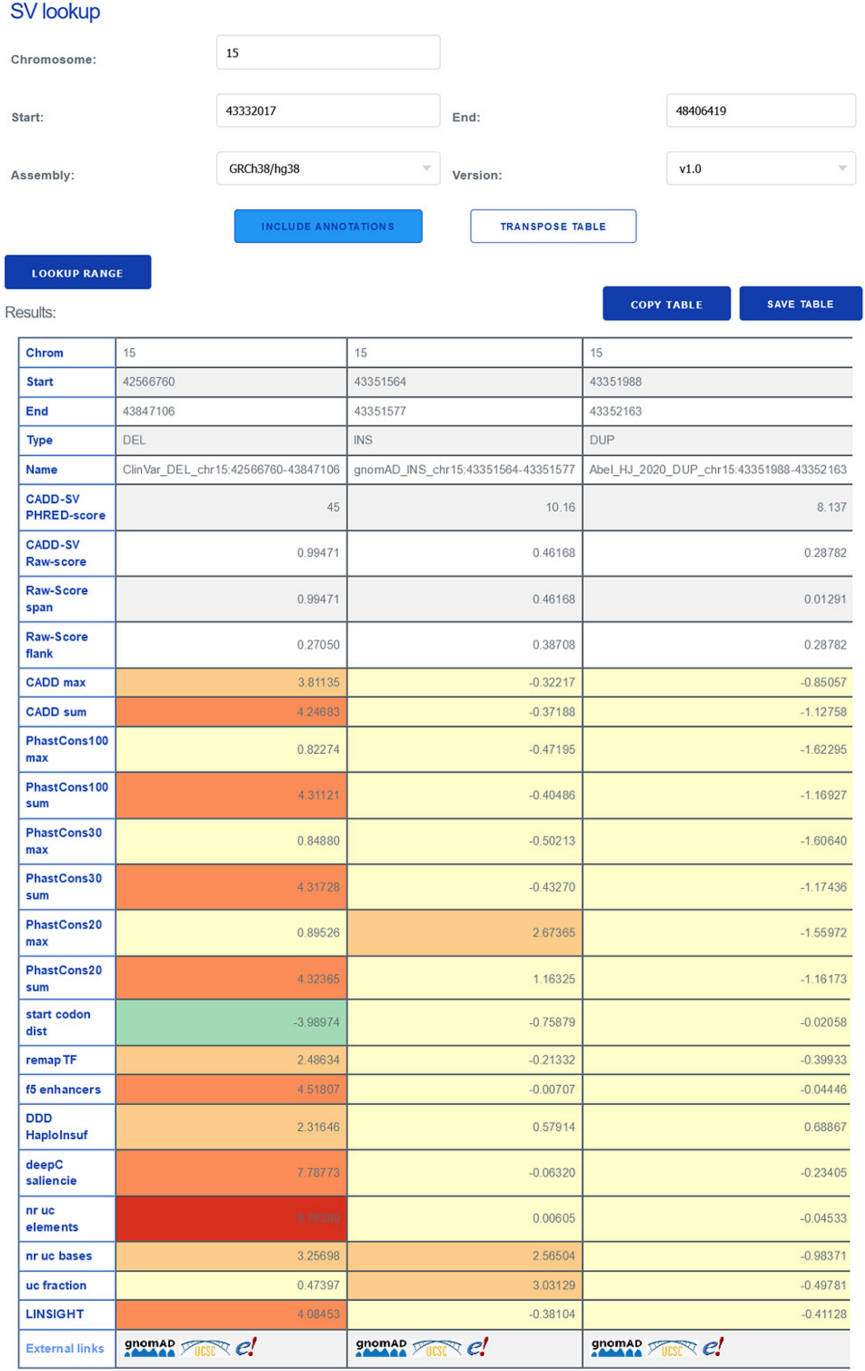
The CADD-SV web server can score custom SV sets, but it can also be used for direct lookup of prescored deletions, duplications, and insertions from gnomAD and ClinVar, as well as call-sets from Abel et al. (2020) and Beyter et al. (2021). For a given SV, the website provides the combined model scores as well as annotation values normalized to the range in the healthy gnomAD cohort (*Z-*score). This enables users to identify interesting variants from color-highlighted extreme feature values and not just by the combined CADD-SV score. Further, the website provides direct links for each SV to external resources like gnomAD, Ensembl, or the UCSC Genome Browser.

To make scores easier to interpret and as outlined above, we Phred-scaled CADD-SV raw scores among all SVs reported in gnomAD-SV. For example, a value of 30 represents that 99.9% of variants reported from healthy individuals score lower than the variant under consideration. In addition, all feature annotations are used and reported after *Z-*score transformation according to the features’ value distribution observed for gnomAD-SV variants. This allows users to inspect the individual features for extreme values easily. For instance, a conservation feature value of four represents an outlier value of four standard deviations away from the gnomAD mean of that specific annotation. Such noticeable values are highlighted by color-coding on the CADD-SV website (Fig. 5) for the prescored variant sets. Generally, CADD-SV scores with or without annotation information are available from our command line tool as well as on the web server for direct variant interpretation. Our online services include region lookups of existing SV data sets, coordinate transfers between human genome builds, the download of prescored data sets and annotations, a simple API for the retrieval of prescored variants, as well as the online scoring of novel SV data sets. Coordinate ranges and variants of other genome builds (i.e., GRCh37/hg19 and NCBI36/hg18) can be used on the web server and are automatically lifted to GRCh38 coordinates (providing the original coordinates in the variant's name field).

## Discussion

We present CADD-SV as an unbiased and powerful tool for the annotation and prioritization of deleterious structural variants. Structural variant calling is prone to biases toward certain types of SVs, as, for example, the signal to detect deletions is vastly different compared to signals of duplication or even inversions (Cameron et al. 2019). Further, the exact annotation of SV breakpoints is often limited, for example, due to their frequent positioning in repetitive sequence (Kosugi et al. 2019). Apart from these universal limitations, changes in the application of arrays and sequencing technologies over the last decades have affected available SV sets. However, in previous work, it seems underappreciated how much of the historic and functional ascertainment is imprinted on potential training and validation sets for machine learning. Specifically, the ClinVar-annotated SVs are comparably large and clustered around well-studied genes. Using an alternative source for the training data, the CADD-SV approach is not confounded and performance can be evaluated broadly, as no allele frequency features nor any ClinVar annotations are included in the features or otherwise considered when building the training sets. The number of labeled SVs to validate the performance of CADD-SV is still limited, however, and assessing the performance on duplications and insertions is limited, as the number of known pathogenic events is small and strongly biased toward coding sequence. We anticipate that future data sets will provide a better opportunity to test and interpret models for duplications and insertions.

Estimating functional effects of SVs is highly complex due to their size (involving different molecular targets) but also due to different mechanistic types of SVs (e.g., deletion, insertion, duplication, or inversion of sequence). Thus, deleteriousness effects cannot just result from the sequence alteration but also from interactions with the sequence context. For example, sequences shielding gene regulation (e.g., TAD boundaries) can be deleted between coding sequences or nonfunctional sequence can be inserted, interfering with an existing regulatory unit. Therefore, we model each SV type (deletions, insertions, and duplications) separately, and we use the sequence span as well as the flanking sequence regions to capture putative pathogenic effects comprehensively. Further, we integrate distance features and a large set of annotations covering both coding and noncoding effects. This allows CADD-SV high predictive performance on known disease variants from ClinVar, which often cover coding sequence and stand out by their gene model annotations and genes scores such as pLI (Lek et al. 2016) or Deciphering Developmental Disorders’ Haploinsufficiency (Firth and Wright 2011). Extending this to other previously described disease mechanisms for pathogenic noncoding variants (Spielmann et al. 2018), CADD-SV makes use of sequence conservation (Siepel et al. 2005), enhancer element annotations (Abugessaisa et al. 2017; Chèneby et al. 2018), and enhancer links (Hait et al. 2018), assay readouts such as RNase-seq or ChIP-seq, as well as information about 3D interactions from the Hi-C directionality index (Schmitt et al. 2016; Calandrelli et al. 2018) or computational predictions such as deepC (Schwessinger et al. 2020).

Inversions and translocations are particularly hard to assess as they are copy number-neutral and their impact is often mediated by proximity of certain functional elements to one another or functional entities such as TADs being broken or reshuffled rather than deleting or inserting functional sequence directly. To our knowledge, there is no training data set sufficient in size and curation to capture the complexity of these events. As no single model could capture the mechanistic diversity of the three currently considered SV types (insertions, deletions, and duplications), CADD-SV reports normalized model scores and features through relative ranks as well as *Z-*scores (i.e., values reported as standard deviations away from the mean) based on SVs from a large cohort of healthy individuals. Phred-scaled model scores provide an intuitive interpretation, and feature normalization enables users to inspect extreme values for the different annotations directly, visually highlighting certain annotations and hinting at potential pathogenic mechanisms beyond the final CADD-SV score. Although designed for genome build GRCh38, CADD-SV can be applied to other genome builds due to an integrated liftOver step of the web server.

In contrast to other tools, length is not a feature of CADD-SV. However, we assume that SV length would be a good indicator of SV impact, as long SVs are more likely to affect coding regions or generally functional annotations. SV length itself might be a confounder too, as long benign SVs might be misinterpreted solely for their length and not for their actual genomic signatures. As the contrasting data sets in the CADD-SV framework are matched in SV length, length as a feature does not contribute to the model. However, some genomic feature transformations, such as the sum of all intersected annotation values or the number of bases above a certain threshold, correlate inevitably with length but are bound to functional annotations being present across the span. AnnotSV (Geoffroy et al. 2018) is powerful and efficient in annotating novel SVs with a wide set of annotations. However, validation of AnnotSV on ClinVar is biased as AnnotSV uses overlap of novel SVs with labeled SVs from ClinVar as a feature. Further, it categorizes SVs in five bins from benign to pathogenic instead of a continuous score. Across multiple data sets, we highlight the increased predictive power of CADD-SV compared to AnnotSV, SVscore (Ganel et al. 2017), and TAD-fusion (Huynh and Hormozdiari 2019). We could only provide a limited comparison to StrVCTVRE (Sharo et al. 2020), which is designed to score only exonic variants. A comparison of SVFX (Kumar et al. 2020) was not possible, as the package is not easily deployed and explicitly normalizes features on a specific training data set. Its released ClinVar variant models are trained on a variant set overlapping with our validation set.

The feature integration implemented by CADD-SV can easily be extended using additional annotations. Currently, we use features derived from experiments conducted in specific cell types (e.g., GM12878, H1, A549, CAKI2). More comprehensive or additional cell types can be included in updated versions. Further, CADD-SV does not make use of the inserted sequence itself. Therefore, future versions of CADD-SV could make use of sequence-based prediction models in addition to reference annotations, for example, to predict open reading frames, repeat content, presence of transcription factor binding sites, or the likelihood of the novel inserted sequence being of open or closed chromatin. This might be powerful in assessing inserted sequence function beyond the surrounding genomic context of the insertion event. In addition, specific mechanistic events such as gene-fusion predictions are not part of our features. CADD-SV can only estimate the effect of such events based on already considered feature values like the distance to genes.

Especially for rare variants, clinical databases like ClinVar or OMIM have incomplete coverage. CADD-SV does not use these databases to derive features as we do not want it to be intrinsically limited to previously known disease genes or to reflect the historic ascertainment that imprints on these databases (Hartley et al. 2018; Haynes et al. 2018). We recognize that computationally distinguishing functional variants from pathogenic variants is difficult and that available curated data sources like ClinVar and OMIM can still be used in downstream interpretation of the results. Evaluating SVs experimentally will provide insights into disease mechanisms that are currently not represented.

In summary, CADD-SV integrates rich sets of annotations in predictive models of SV effects. CADD-SV is built from machine learning models with an unbiased training using evolutionarily derived and putative benign variants that underwent millions of years of purifying selection. These variants are contrasted with a background set of the same size and length, encountering deleterious events by chance. We show that our approach is able to model and score deletions, insertions, as well as duplications, and we validate the CADD-SV models using clinically annotated, noncoding, or population germline SVs as well as somatic SVs reported in cancer patients. To highlight the potential of CADD-SV, we applied our tool to functional SVs identified from selection screens, QTL studies, or variants identified across many, supposedly healthy individuals. Most of the top-scored variants in the healthy population data set are singletons, suggesting purifying selection on these SVs, and we were able to pinpoint pathogenic variants in multiple cases.

## Methods

### Training data set

We use a set of previously identified evolutionarily fixed chimpanzee and human-derived SVs (Kronenberg et al. 2018) and refer to the autosomal human and chimpanzee deletions and insertions from this set as proxy-neutral or proxy-benign. A set of randomly distributed SVs over the human autosomes was obtained by shuffling the ape SVs matched in length and number (within coordinates considered alignable by Kronenberg et al. 2018). We refer to this set as proxy-deleterious. To compare these SVs with those in ClinVar (Landrum et al. 2018), we annotated them with the distance to the next start codon, and pLI and haploinsufficiency scores (Supplemental Fig. 1). We use sets of variants derived from human and chimpanzee to score different SV types. For novel human deletions, we chose the chimp deletions to model the span and human deletions to model the SV flank. Respective annotations are present along the range of chimpanzee deletions in the human genome build, although they are absent for derived human deletions. Similarly to score insertions, we use the derived human insertions to model the flank and the chimpanzee insertions to model the site of an insertion (see Fig. 1A). Duplication sites are modeled by the chimpanzee deletion model for the span and human insertion model for the flank, as the span of duplications contains known sequence most similar to the one found in annotated deletion sequences. Sex chromosomes were not used in the training data set as the quality of X and Y Chromosomes, especially on a comparative level, is still poor. CADD-SV scores variants on X and Y by applying the autosomally derived model, but we recommend to be cautious in the absolute comparison of scores.

### Feature annotation and transformation

We obtained a set of 127 continuous human-derived features (see Supplemental Table 1) ranging from species conservation, distance to gene model hallmarks, over to genome architecture features such as the directionality index derived from Hi-C data sets. We use customized bash and R scripts to annotate the contrasting SV sets using BEDTools (Quinlan and Hall 2010) and tabix (Li 2011). All features are *Z*-score (mean 0, variance 1) transformed using 20,000 randomly selected SVs of the same-type from the gnomAD-SV release v2.0 (Collins et al. 2020). By doing so, we allow the user to immediately see feature values from the annotated SVs that correspond to extremes in the gnomAD-SV set, although this SV set itself is not impacting our models, as the same transformation is applied for both training class labels. Further, a feature value being extreme in the gnomAD-SV set is also unrelated to how this feature is used in the model (i.e., feature importance for RFs or coefficients in the case of linear models). All SVs are annotated over the span of the primarily affected sequence (span) as well as 100 bp up- and downstream of the site of the structural rearrangement (flank) (see Fig. 1C). Predictions based on the flanking sequences are generally dependent on the exact identification of breakpoints. However, experimentally validated breakpoints for our training data set do not exist, and even with the most advanced SV typing approaches, blurry breakpoint annotations will still exist for repetitive sequence contexts. Therefore, including distance information for functional annotation in the model provides a more flexible approach. From the different annotations, we create summary statistics and transformations as model features. These are summarized in Supplemental Table 1. The annotation framework automatically retrieves the features from primary annotation sets using the workflow management system Snakemake (Köster and Rahmann 2012). It tabulates results in a BED-like format that is used in the CADD-SV model. Missing values are imputed with zeros.

### Models

We trained logistic regression and random forest classification models contrasting proxy-benign and proxy-deleterious training data sets. Models are trained in R (v3.5.1) (R Core Team 2021) for the SV spanning regions for deletions and duplications and the site of integration for insertions (span), as well as 100 bp up- and downstream of the reported breakpoints (flank) (Fig. 1C). For logistic regression, we use the R generalized linear model implementation, and for random forests, the package “randomForest” (Liaw and Wiener 2002). For random forests, we limit the number and depth of the decision trees based on a hyperparameter search (explored ranges for ntree = {25, 50, 75, 100, 200, 500, 1000}, nodesize = {10, 50, 100, 250, 500, 1000}, maxnodes = {10, 50, 100, 250, 500, 1000}, whereas one parameter was optimized, the other parameters were set to 100) (Supplemental Fig. 13). We randomly withheld 10% of the annotated SVs as holdout and assessed model performance metrics using the R Package PRROC (Grau et al. 2015).

### CADD-SV scoring

Each novel SV is annotated along the span and 100-bp flank region and scored using the span and flank models of the respective SV type. The max (more pathogenic) output score of each model is used as the CADD-SV raw score and included in the output. Additionally, a Phred-scaled (−10 log_10_) CADD-SV score is reported for each raw score from the relative rank of the variant's score in the gnomAD-SV score distribution of the same type of SV. We opted against relative ranking of SVs according to gene density, allele frequency, or SV size (see Supplemental Material; Supplemental Fig. 14) and only separate them by SV type. Instead, we provide an additional relative ranking according to a putative healthy population cohort, represented by a Phred score. These Phred scores range from 0 to 48, with a value of 20 corresponding to the top 1% and a value of 30 corresponding to top 0.1% of the scores observed for gnomAD-SV. Higher CADD-SV scores therefore indicate a larger proportion of potentially pathogenic variants.

### Model validation

CADD-SV was designed to be unaffected by known biases found in clinically curated data sets such as ascertainment biases in the choice of genes to be studied. It does not use curated SV sets in training, it does not derive features from clinical data sets such as ClinVar or OMIM, and it does not use gnomAD-SV allele frequencies as features either. Therefore, CADD-SV can be validated using those data sets. Between sets, SVs were not matched by size, frequency, or gene density. As outlined above, CADD-SV makes use of all features independent of the specific SV size or gene density. SV allele frequency is explicitly not part of the model. We are confident that this enables us to score short, gene-poor, pathogenic SVs as well as long nonfunctional SVs appropriately.

Pathogenic and benign annotations for clinical SVs (Landrum et al. 2018) were downloaded from ClinVar (https://www.ncbi.nlm.nih.gov/clinvar) on June 24, 2021. Only variants with pathogenic or benign labels of at least 50-bp-length and annotated as deletion (pathogenic n = 3262, benign = 33), duplication (pathogenic n = 82, benign n = 4), or insertion (pathogenic n = 78, benign n = 18) are considered. Further, to increase the number of pathogenic insertions, unique pathogenic insertions (n = 39) reported by Hancks and Kazazian (2012) and Gardner et al. (2019) were added. Area Under the Receiver Operating Characteristic metrics are calculated using the PRROC R-package (Grau et al. 2015).

Germline SVs identified from healthy individuals over various populations (Collins et al. 2020) were downloaded from gnomAD-SV release v2.0 (https://gnomad.broadinstitute.org/downloads). Allele frequency values of common and ultrarare SVs are determined across all available populations. Common variants are defined as minor allele frequency >0.05, and ultrarare variants are defined as singletons. To show the clinical benefit of prioritization of SVs using CADD-SV, we use 1000 Genome genotyped SVs (The 1000 Genomes Project Consortium 2015) and add one (randomly selected) labeled pathogenic SV found in ClinVar into the reported set of individual specific SVs. From the 1000 Genome SV events, we consider *Alu* and LINE-1 SVs to be insertions. We report the rank of the pathogenic SVs within the complete SV sets.

Somatic SVs (n = 95,749) from cancer patients were obtained from the International Cancer Genome Consortium (Campbell et al. 2020) at https://dcc.icgc.org/api/v1/download?fn=/PCAWG/consensus_sv/final_consensus_sv_bedpe_passonly.icgc.public.tgz. In addition, insertions reported in cancer genomes were taken from Qian et al. (2017) (n = 18). To assess the performance of CADD-SV beyond coding regions, we use noncoding SVs (n = 687) that are known to impact human gene expression in data from the GTEx Consortium (Chiang et al. 2017).

To assess CADD-SV's ability to prioritize functional stretches of DNA, we used healthy population SVs from gnomAD-SV containing a genome-wide association study-linked SNV. We assume that presence of an association with a functional trait can be seen as a proxy for functional SVs. The GWAS catalog was downloaded from https://hgdownload.soe.ucsc.edu/goldenPath/hg38/database/gwasCatalog.txt.gz. Further, we use deletions and insertions reported to be associated with changes in gene expression patterns as well as SVs under natural selection (Ebert et al. 2021), both hinting toward functional stretches of DNA that are beyond coding effects.

### SV scoring tools

CADD-SV performance on various validation sets was compared to existing tools SVScore (Ganel et al. 2017), AnnotSV (Geoffroy et al. 2018), StrVCTVRE (Sharo et al. 2020), and the TAD-fusion score (Huynh and Hormozdiari 2019) using standard parameters. TAD-fusion only scores deletions and was primarily developed to identify 3D genome alteration. As SVScore and TAD-fusion scores were not available for the current genome build GRCh38, UCSC liftOver (Kuhn et al. 2013) was used to transfer SV coordinates and respective scores.

### Implementation

Novel SVs can be scored with a pipeline implemented in Snakemake (Köster and Rahmann 2012), using conda (Grüning et al. 2018) for dependency management. CADD-SV was designed to be applicable for bioinformaticians and clinicians alike. The source code for the framework is available for download on GitHub (https://github.com/kircherlab/CADD-SV/). Conda and Snakemake guarantee easy installation procedures as well as stability through dependency management. Further, we implemented CADD-SV to be time- and memory-efficient, while being highly parallelizable for application on a cluster-network. A set of 1000 short SVs can be scored on a regular laptop in 13 min using 600 MB of memory (Supplemental Fig. 15). However, in contrast to all competing tools, CADD-SV jobs are highly parallelizable, strongly improving time-performance. In addition to the source code, a web service (https://cadd-sv.bihealth.org/) allows for online scoring of SVs in a BED-like format as well as for obtaining results for different human genome builds (GRCh38; NCB16 and GRCh37 through automated coordinate liftOver). In addition, prescored variants from cohorts such as gnomAD or ClinVar can be queried online including all feature annotations. For better interpretability, feature outlier values are color-coded based on their *Z-*scores.

### Software availability

CADD-SV prescored variant sets as well as a website for the interpretation of novel deletions, insertions, and duplications are available at the CADD-SV web server (https://cadd-sv.bihealth.org/) as well as Zenodo (https://doi.org/10.5281/zenodo.5963396). The CADD-SV framework can be cloned and used from GitHub (https://github.com/kircherlab/CADD-SV/) and is available as Supplemental Code. All external data sets used are publicly available under the locations specified in the Methods.

## Supplementary Material

Supplemental Material
