# Supplementary material for: A framework to score the effects of structural variants in health and disease
Source: Genome Res. 2022 Apr;32(4):766–77. doi: 10.1101/gr.275995.121 (PMC8997355; doi:10.1101/gr.275995.121)
Supplement: Supplemental Material [file supp_32_4_766__DC1.html]

A framework to score the effects of structural variants in health and disease — Supplemental Material 

# A framework to score the effects of structural variants in health and disease

## Supplemental Material

- Supplemental\_Code.zip
- Supplemental\_Material.pdf
- Supplemental\_Tables\_1-3\_.xlsx
