# Supplementary material for: A framework to score the effects of structural variants in health and disease
Source: Genome Res. 2022 Apr;32(4):766–77. doi: 10.1101/gr.275995.121 (PMC8997355; doi:10.1101/gr.275995.121)
Supplement: Supplemental Material [file supp_gr.275995.121_Supplemental_Material.pdf]

Supplemental Material

A framework to score the effects of structural variants in health and disease

Philip Kleinert[1], Martin Kircher[1,2]

[1] Berlin Institute of Health (BIH) at Charité – Universitätsmedizin Berlin, Berlin, Germany
[2] Institut für Humangenetik, Universität zu Lübeck, Lübeck, Germany

Supplemental Methods

Relative ranking of CADD-SV score

As CADD-SV scores depend on the annotated features for each SV, a relative ranking depending on certain feature groups could be applied. For instance, variants could be relatively ranked with SVs that are of similar size, allele frequency or gene density. CADD-SV does not implement such relative ranking, instead we are ranking the raw model scores relative to the biggest and least biased population SV call set available to us (gnomAD-SV). We do that for the following reasons:

First, CADD-SV makes use of features that are independent of variant size like GC content, fraction of conserved variants or coding sequence. In addition, as CADD-SV was specifically developed to score SVs independent of SV length (which is not a feature in the model and the training dataset is matched in SV length), we are convinced that features that correlate with SV length like the number of enhancers or coding sequence being affected are part of the signal that makes CADD-SV powerful. CADD-SV is therefore able to score short functional SVs as well as long SVs effectively. In other words, we assume that SV length is part of the signature of pathogenic SVs as they are more likely to interfere with functional sequence, however unlike other tools we do not make use of length directly, but indirectly by the higher likelihood of overlapping with a functional DNA stretch.

Second, variant frequency is difficult to use, as most novel SVs will not have a population frequency. Further, allele frequency is not part of the training dataset as by design the fixed variants have a frequency of one, while the matched randomly drawn variants will most often not overlap with existing variants and therefore have a frequency of zero. This would also bring up the challenge of (fuzzy) matching variants to a reference panel to retrieve potential allele frequencies and potentially bias the score outcome for call-sets with varying breakpoint precision.

Third, SVs normalized by gene density would most definitely put more weight on non-coding SVs or other genomic feature sets. However, as we tried to design CADD-SV as free from specific human biases as possible, we consider gene density as an important value to score for function. We designed the score to be genome-wide without a pre-designed focus on certain genomic regions.

Finally, we would like to stress that we are providing the Phred-scaled CADD-SV score in addition to the raw model score as a direct output. Even though we recommend using the ranked version of the CADD-SV score for easier interpretation, users worried about using a relative ranking to gnomAD-SV cohort can use the raw score.

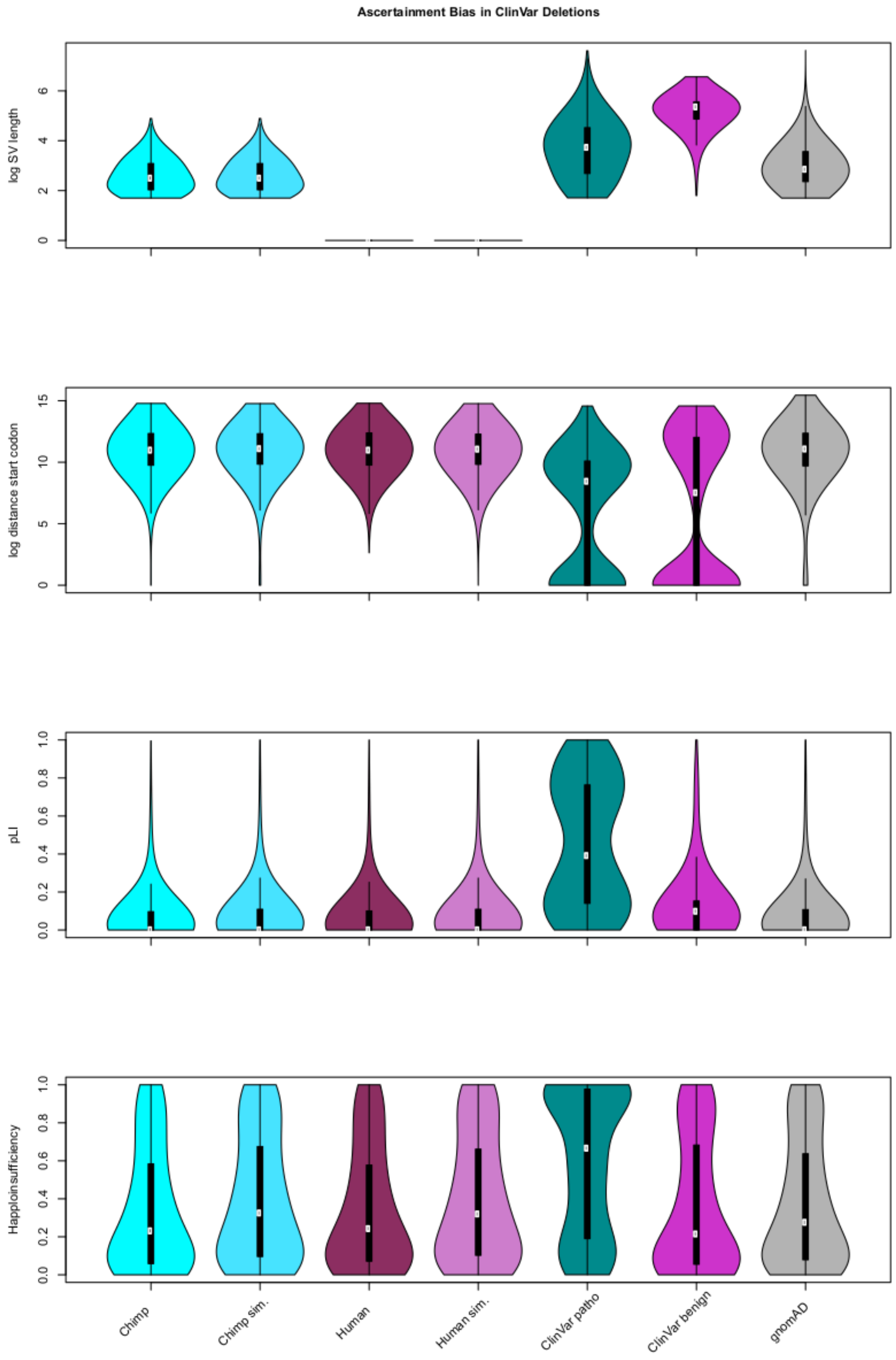

Supplemental Figure 1

Ascertainment bias in labeled deletion datasets. To make accurate predictions using machine learning it is crucial to have an unbiased dataset to train on. ClinVar pathogenic or benign labelled deletions are hand curated and individually verified but are biased towards very large deletions and are clustering around well-studied genes (as shown in the excess of high pLI and Haploinsufficiency scores). Our evolutionary derived dataset however does not suffer from these kinds of ascertainment bias and is similar to the occurrence of deletions in a healthy population cohort (gnomAD-SV v2.0).

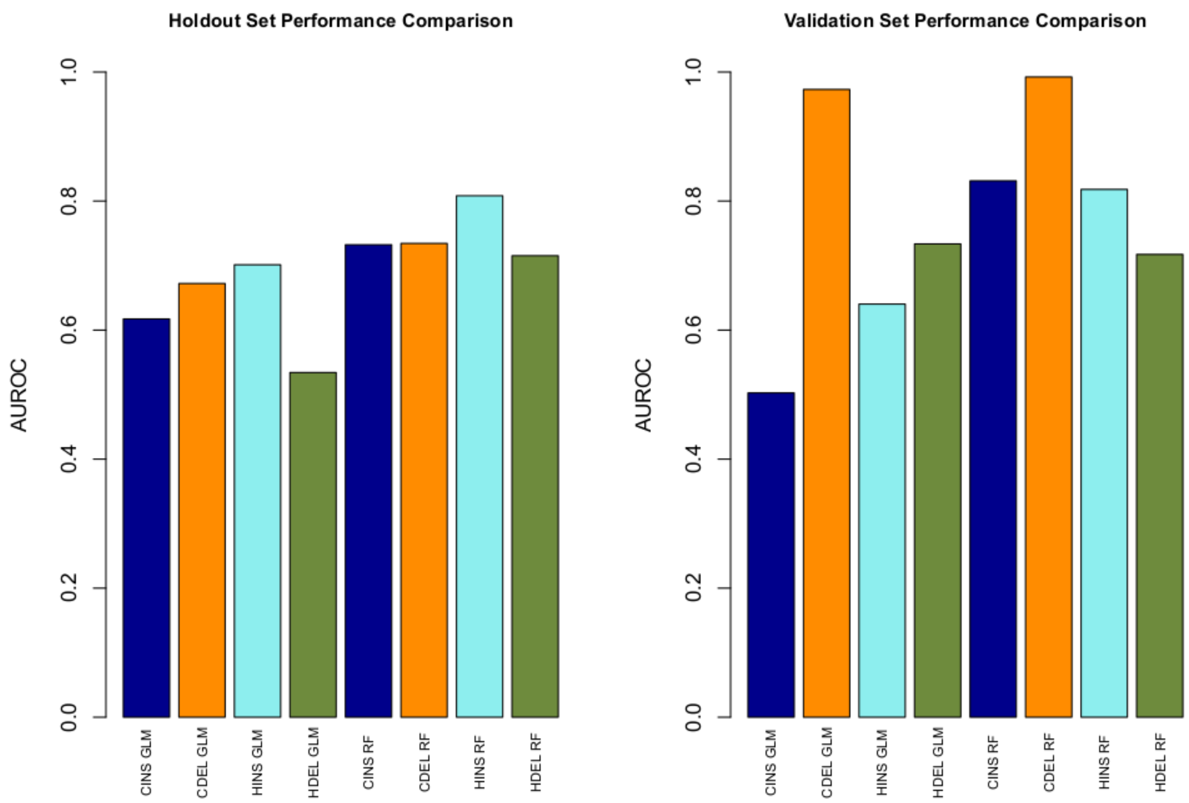

Supplemental Figure 2
Model comparison of Random Forest classifiers and generalized linear models trained using the R GLM package. We validated the performance of both classifiers using 10% randomly sampled holdout data (left) as well as one of the validation sets (labelled pathogenic SVs from ClinVar vs. SVs in gnomAD, right).

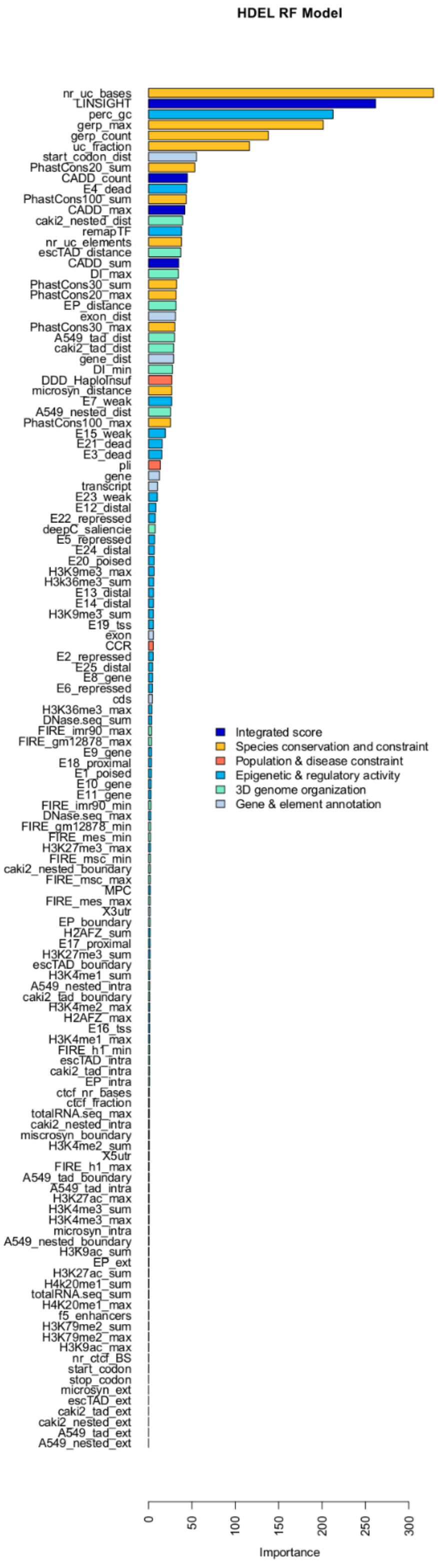

Supplemental Figure 3
Feature contributions of the human deletion (human DEL) flank model.

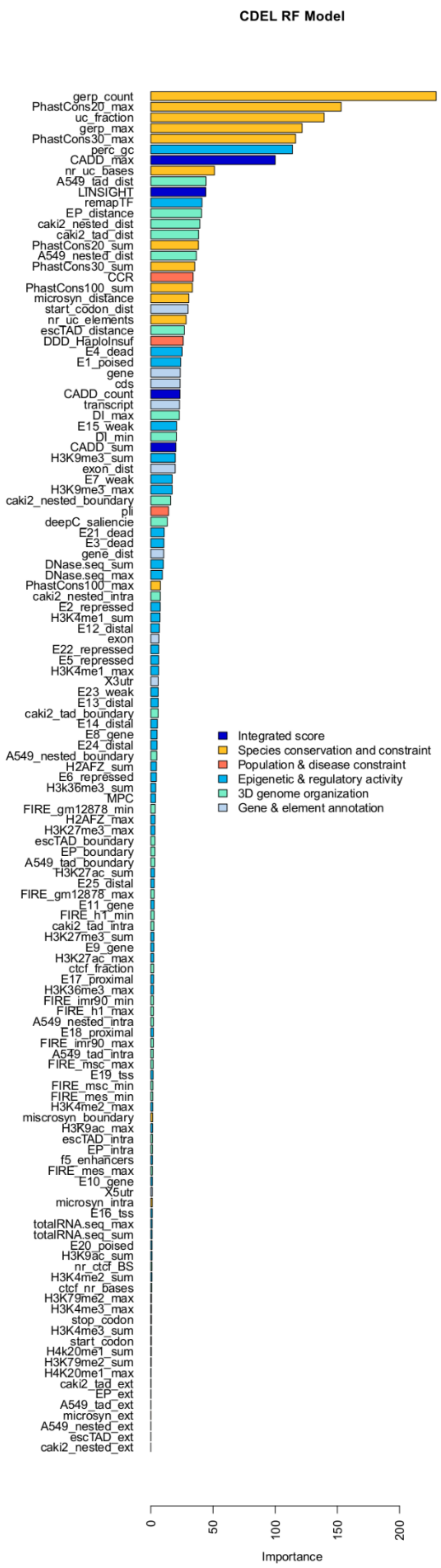

Supplemental Figure 4

Feature contributions of the chimpanzee deletion (chimp DEL) span model.

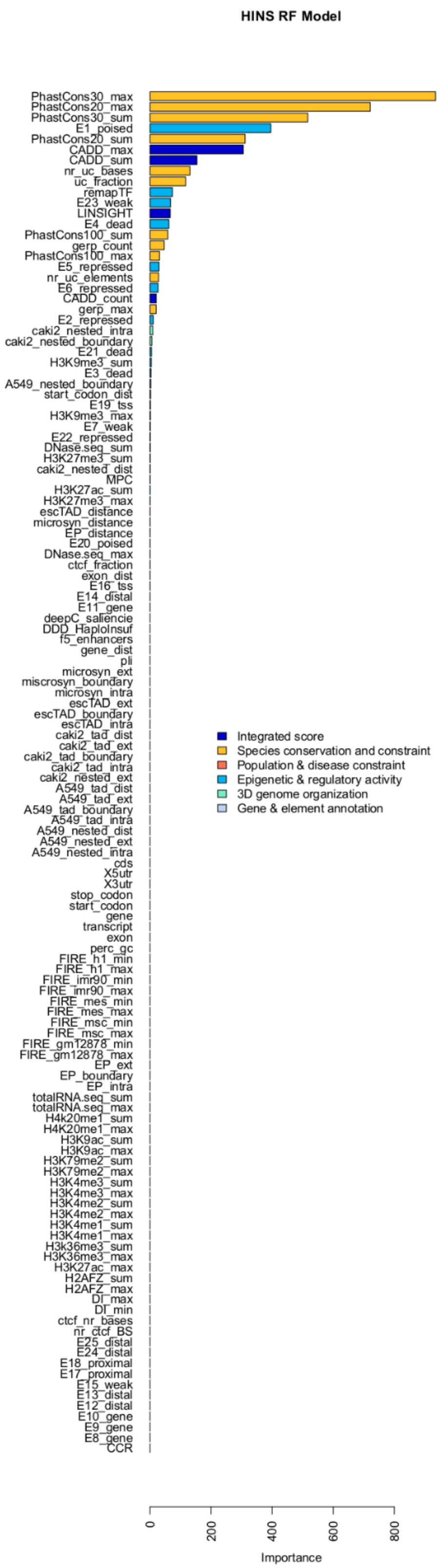

Supplemental Figure 5

Feature contributions of the human insertion (human INS) flank model.

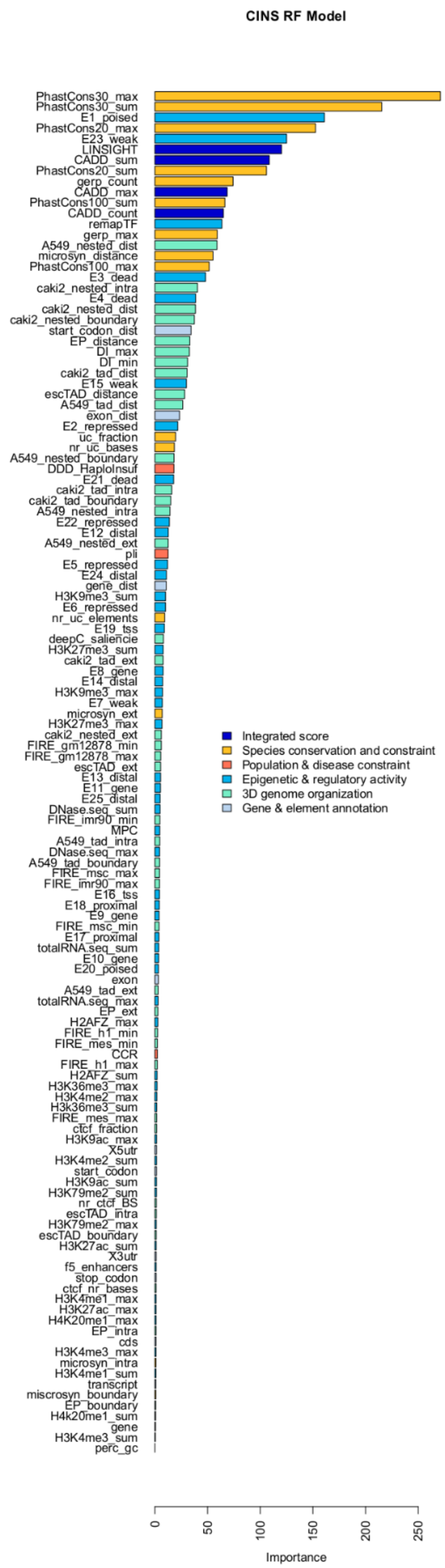

Supplemental Figure 6

Feature contributions of the chimpanzee insertion (chimp INS) span/site model.

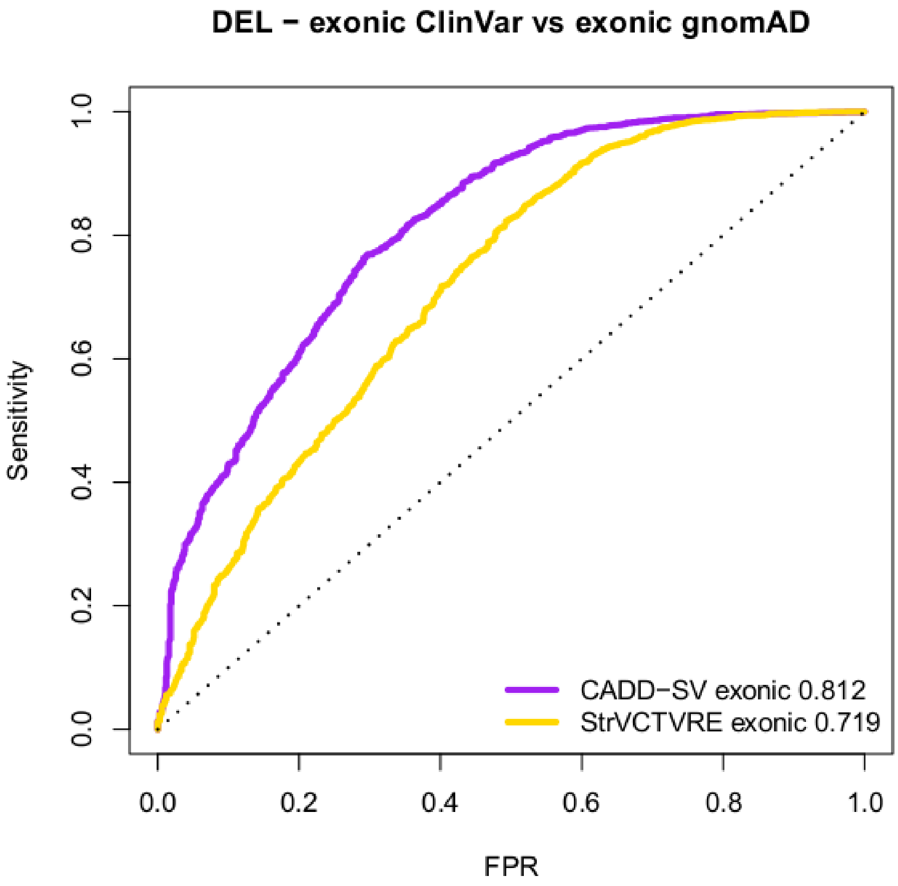

Supplemental Figure 7
CADD-SV and StrVCTVRE performance compared on ClinVar exonic sequences vs gnomAD-SV common exonic SVs.

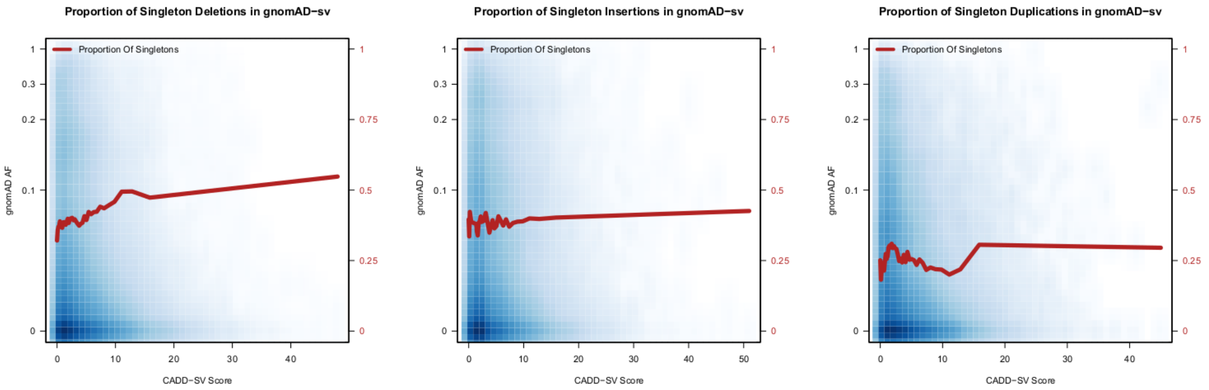

Supplemental Figure 8

Proportion of singleton insertions and duplications in the gnomAD-SV data set of putative healthy individuals. The pathogenic CADD-SV score tail (≥20, being the top 1% most pathogenic) is enriched in singletons, suggesting purifying selection against SVs with high CADD-SV scores. However, this effect is less pronounced in insertions and duplications compared to the deletions.

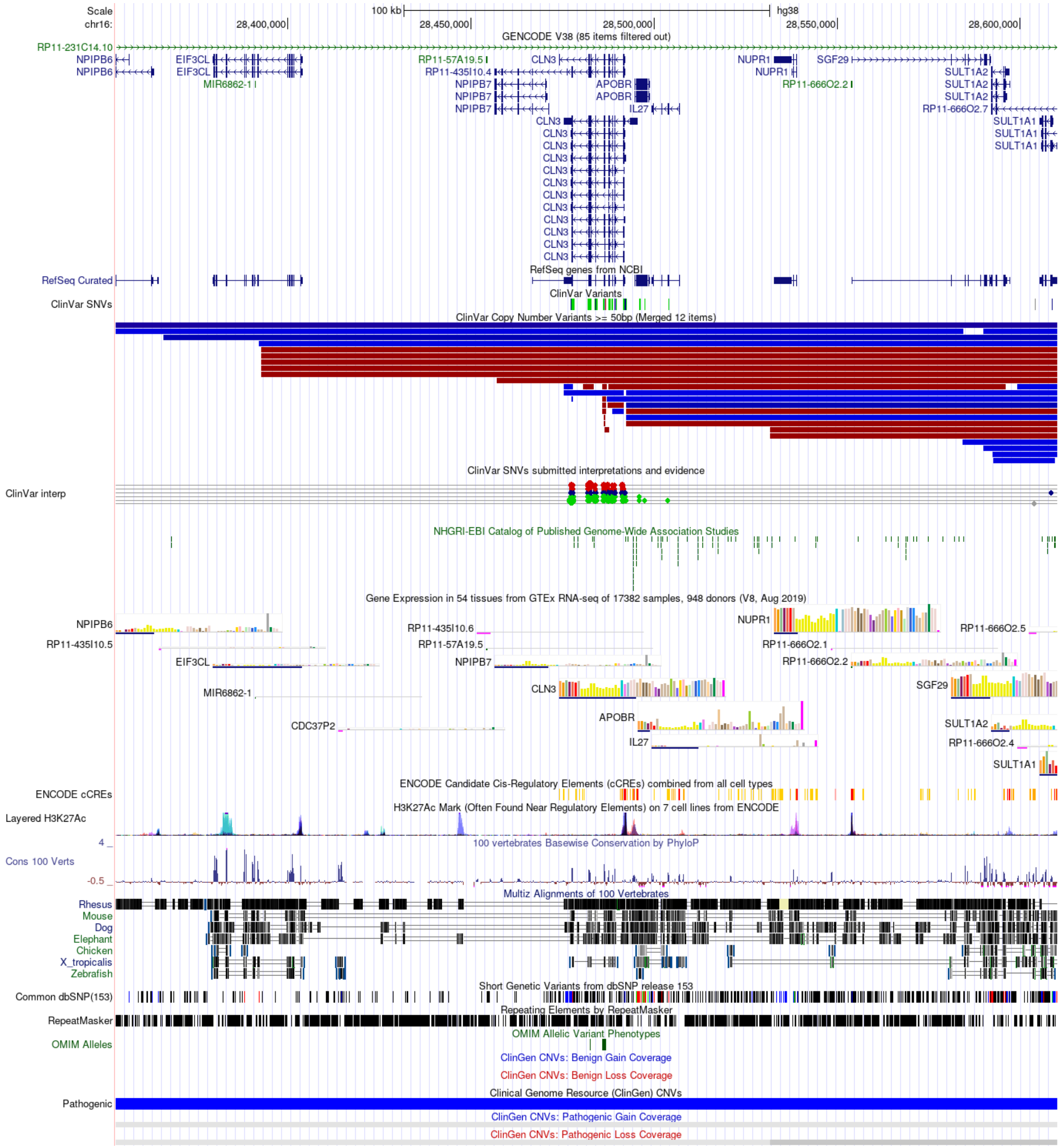

Supplemental Figure 9

UCSC Genome Browser tracks of a region (chr16:28353000-28610100) deleted in two individuals present in the gnomAD-SV cohort. Various genes are affected, with *CLN3* being identified as causing Batten disease, a fatal disease of the nervous system. Various positions of this SV are highly conserved among 100 Vertebrate Genomes, giving CADD-SV power to detect this SV with a high score. This SV is not a singleton, suggesting a recessive disorder. In some cases, Batten disease can also have a late onset of disease symptoms, potentially explaining the presence of this SV in a healthy cohort.

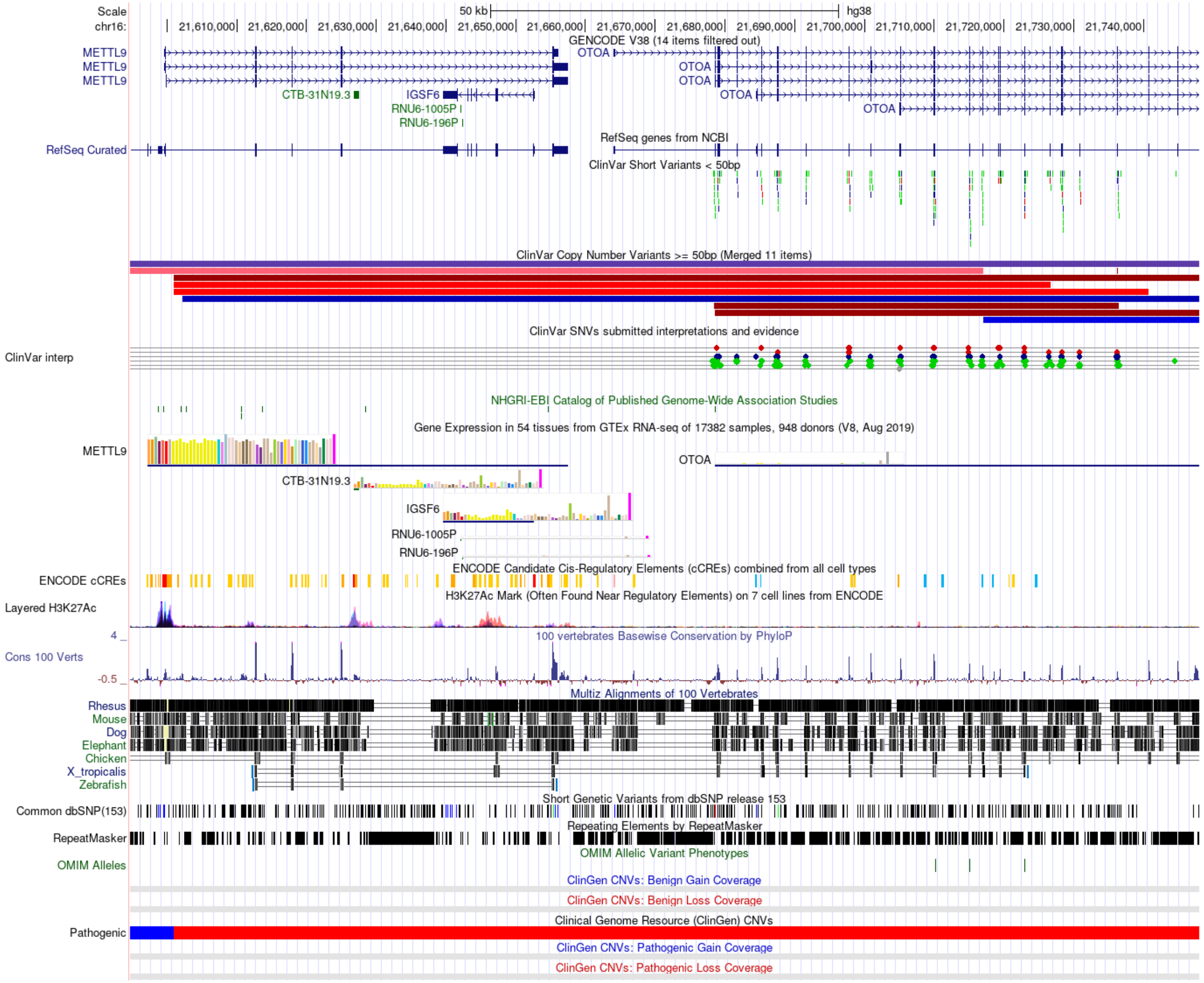

Supplemental Figure 10

UCSC Genome Browser tracks of a region (chr16:21594700-21748000) deleted in 20 individuals present in the gnomAD-SV cohort. Two genes are affected, with *OTOA* being identified as autosomal recessive disease causing severe hearing loss, when rendered dysfunctional by ClinVar annotated SNVs within the *OTOA* gene body. Further, various positions of this SV are highly conserved among 100 Vertebrate Genomes, giving CADD-SV power to detect this SV with a high score. Unlike other putative pathogenic SVs, this SV is not a singleton, suggesting a recessive disorder or reduced purifying selection on phenotypes such as hearing loss

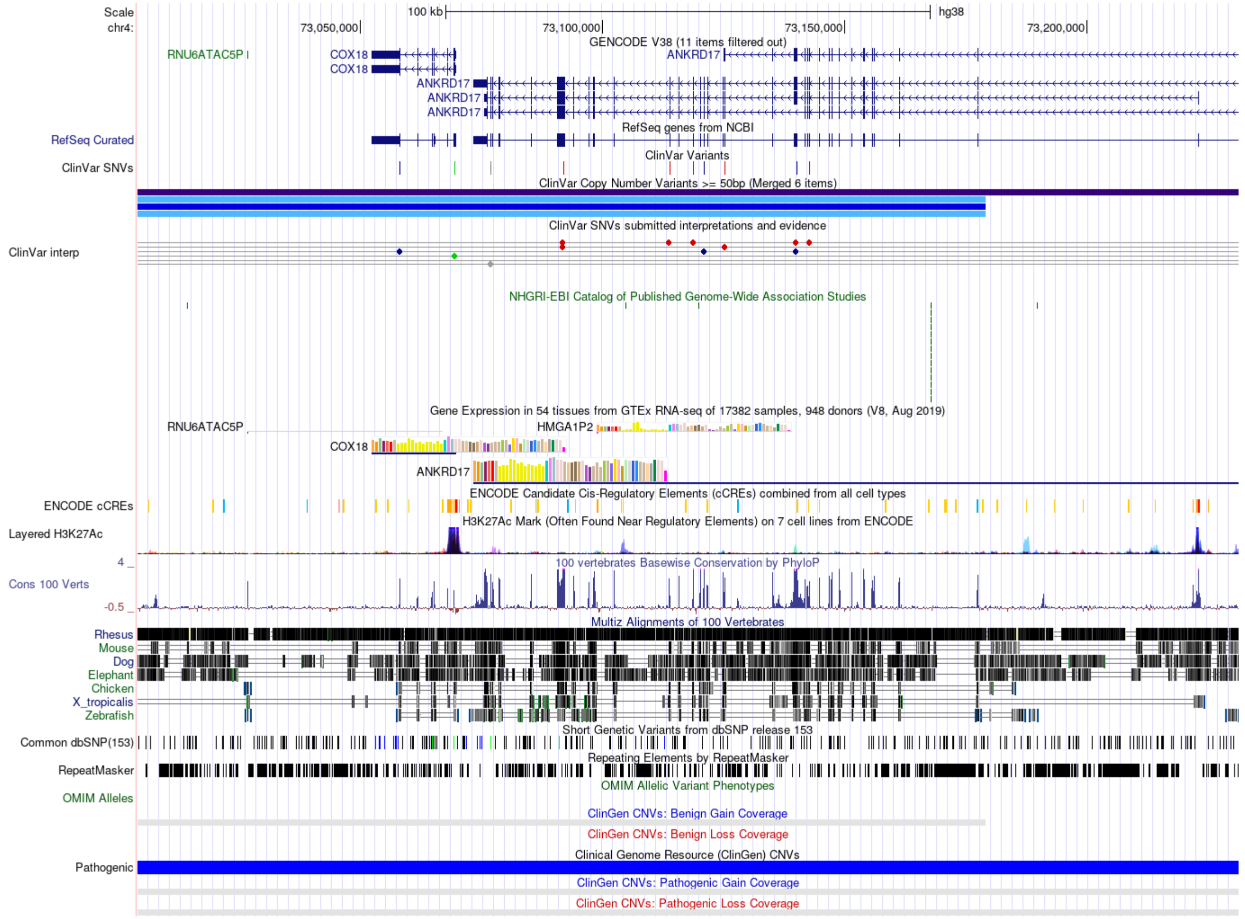

Supplemental Figure 11

UCSC Genome Browser tracks of a region (chr4:73004055-73231324) deleted in one individual present in the gnomAD-SV cohort. Two genes are affected, with *ANKRD17* being identified as autosomal dominant disease causing Chopra-Amiel-Gordon syndrome (CAGS) with various pathogenic ClinVar SNVs being annotated within the gene body of this gene. CAGS patients are characterized by developmental delay and intellectual disability ranging in severity from moderate to severe. Various positions of this SV are highly conserved among 100 Vertebrate Genomes, giving CADD-SV power to detect this SV with a high score.

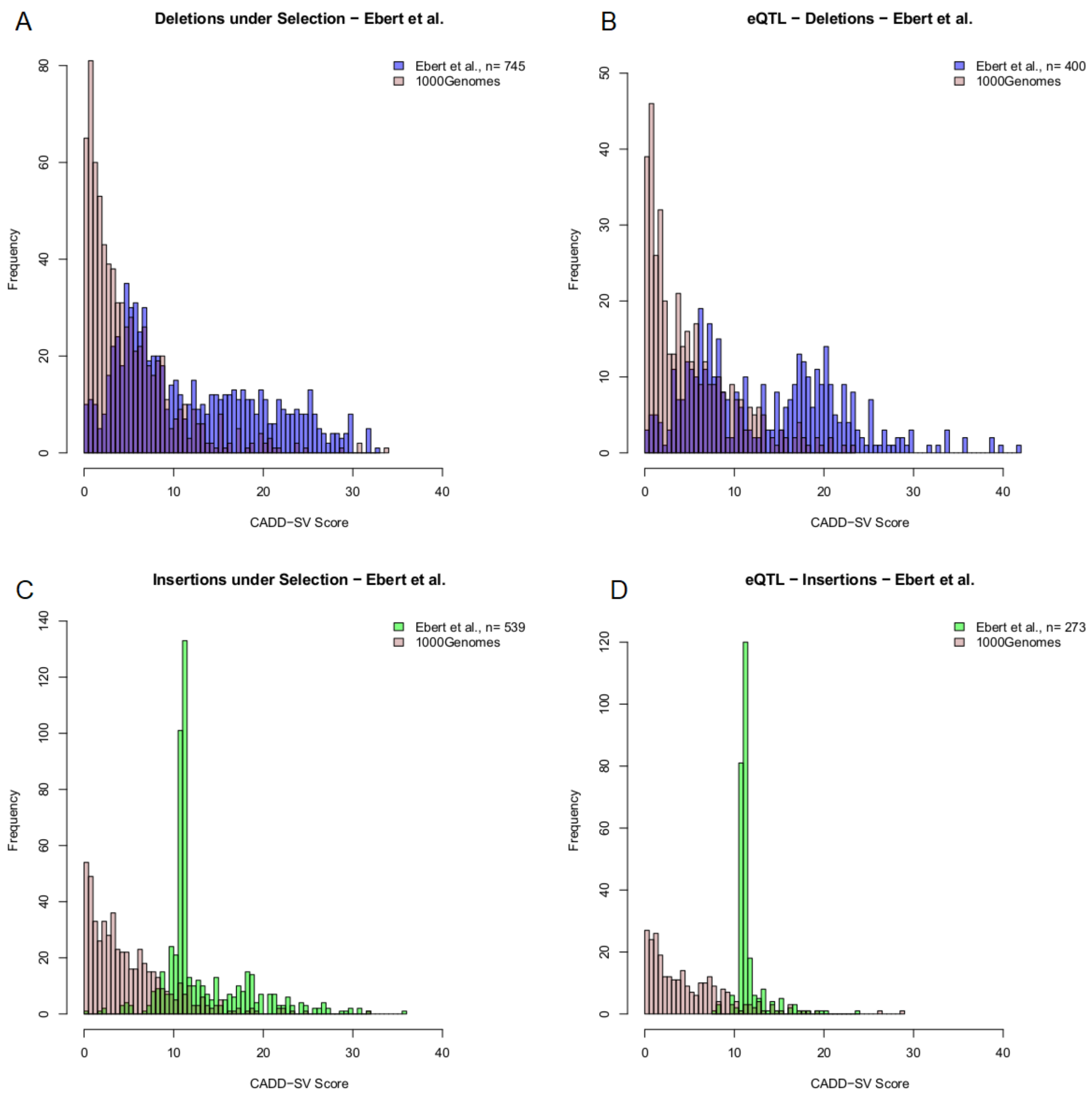

Supplemental Figure 12

Functional deletion and insertion SVs annotated in Ebert et al. 2021. CADD-SV prioritizes both SVs under natural selection as well as expression associated SVs in this data set. Shown are score distributions for the functional set (deletions in blue, insertions in green) against the same number of randomly drawn SVs from the 1000 Genomes project. Note that CADD-SV is a Phred-scaled score distribution with high values corresponding to high pathogenicity.

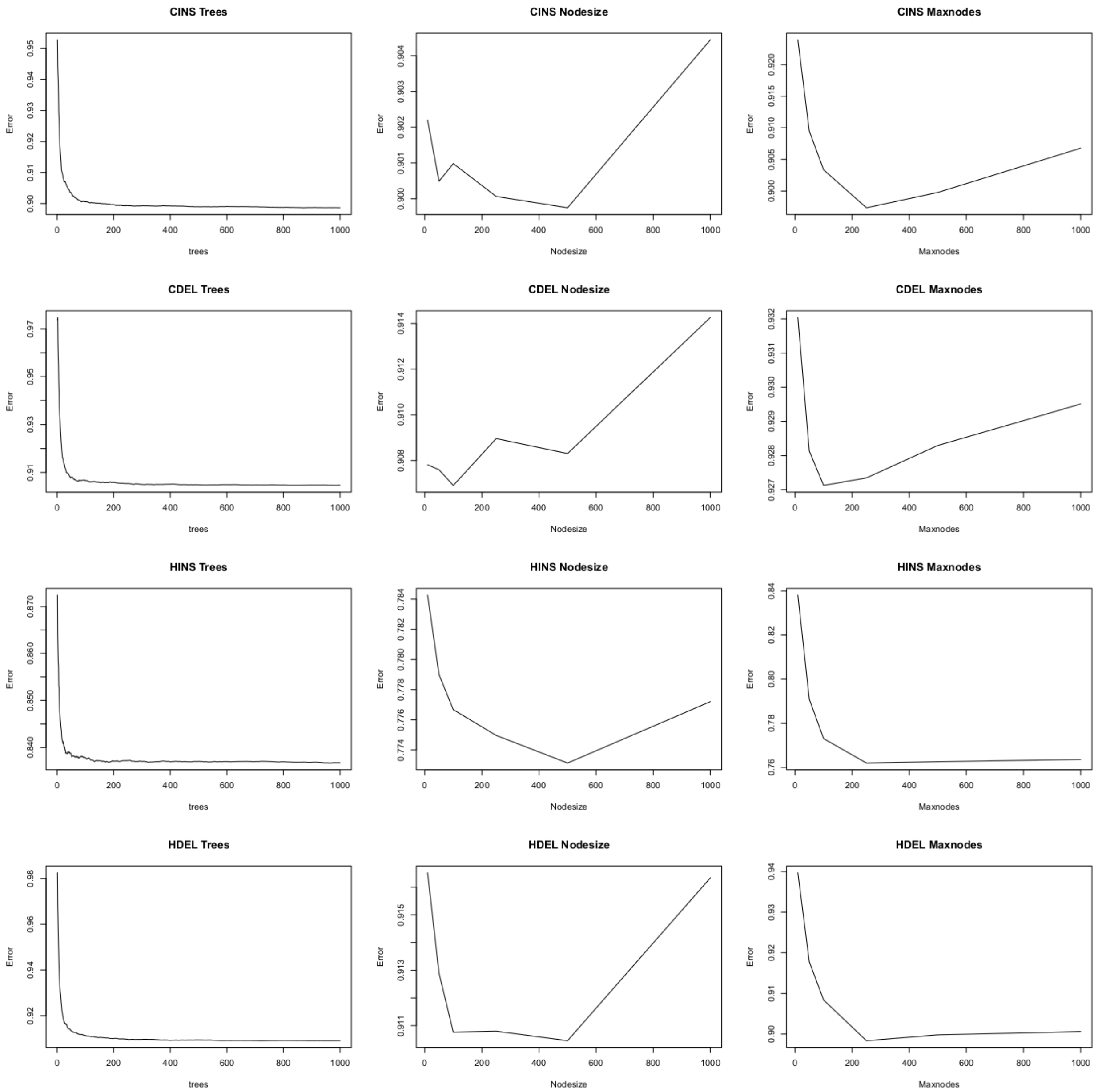

Supplemental Figure 13

Hyperparameter search for all Random Forest models. We explored the mean of squared errors for the parameters "nodesize" and "maxnodes" for both values of n= {10, 50, 100, 250, 500, 1000} and chose parameters minimizing error and overfitting. The number of trees (ntree = {25, 50, 75, 100, 200, 500, 1000}) for each model was chosen based on observing no further improvement of error by increasing the number of trees. CINS: Chimp Insertions, CDEL: Chimp Deletions, HINS: Human Insertions, HDEL: Human Deletions

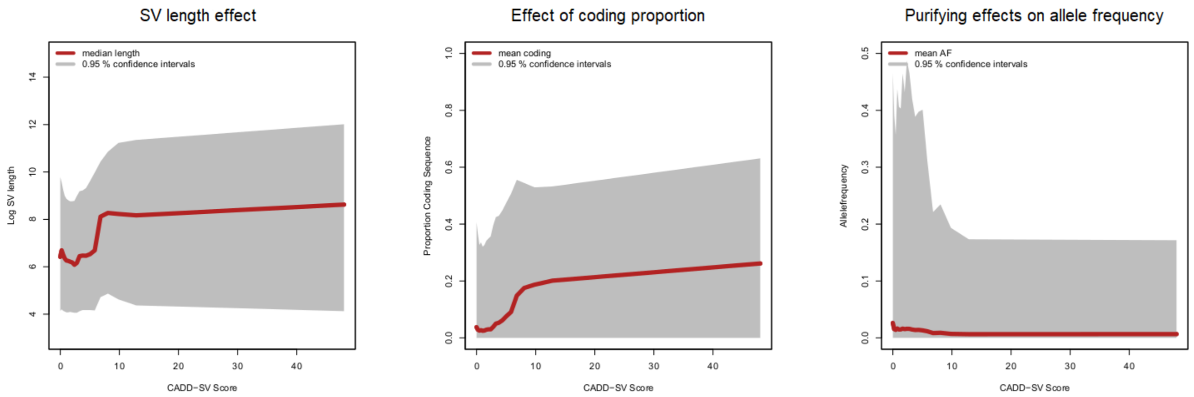

Supplemental Figure 14

CADD-SV score distribution as a function of length, proportion of coding sequence and allele frequency. While high CADD-SV scores are enriched in longer SVs that are more likely to be coding, the pathogenic tail (≥20, being the top 1% most pathogenic scores) is depleted in common SVs. Note that the Phred-scaled CADD-SV score (log_{10} scale) is used with high values corresponding to high pathogenicity.

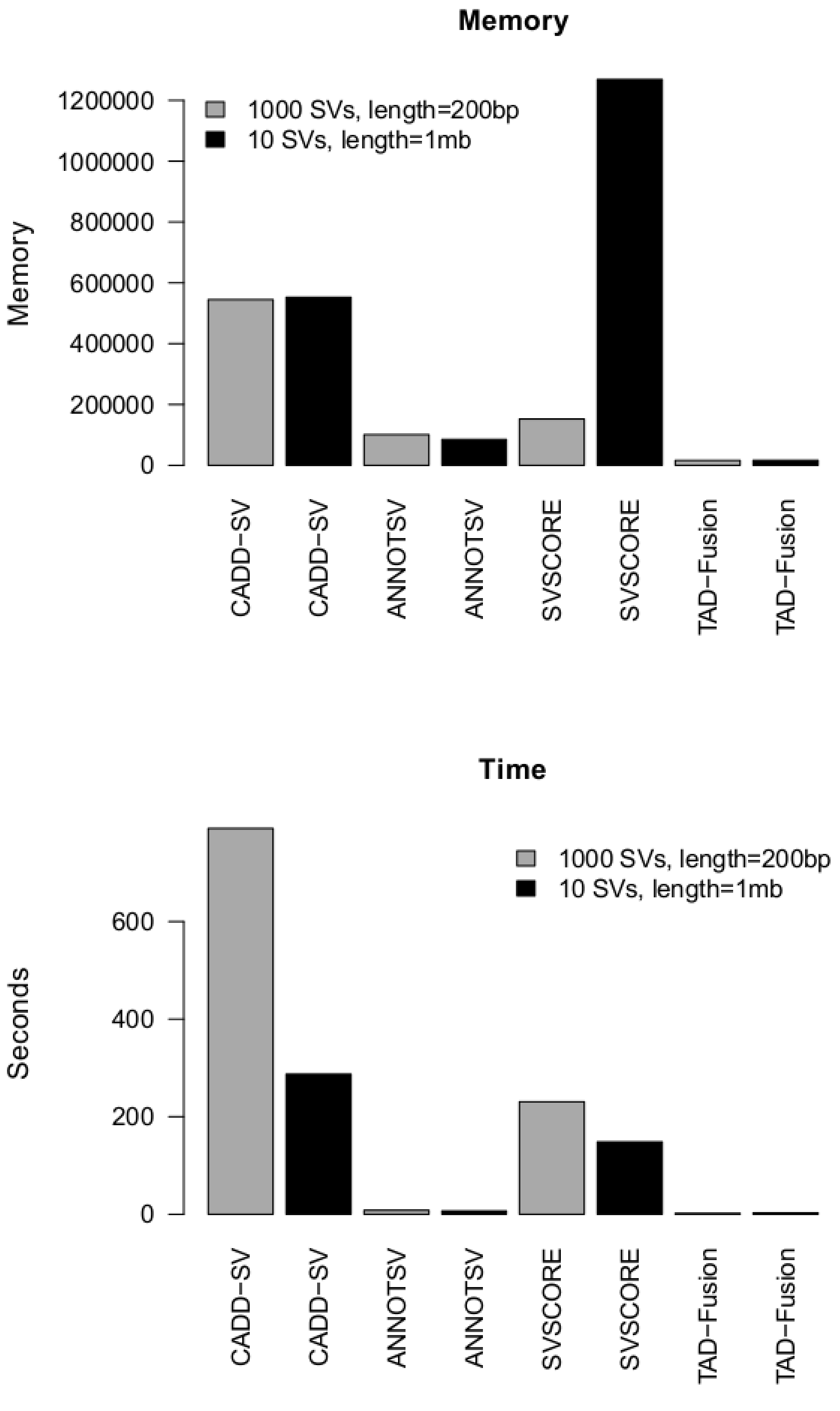

Supplemental Figure 15

CADD-SV time and memory consumption compared to competing tools. CADD-SV annotates and transforms a wide set of comprehensive features, which is time and memory intensive. However, due to its small memory footprint, it is feasible to run CADD-SV on regular laptops. When run in high performance clusters environment, CADD-SV profits from a high degree of job parallelization.
